# A *Trypanosoma cruzi* zinc finger protein that is implicated in the control of epimastigote-specific gene expression and metacyclogenesis

**DOI:** 10.1017/S0031182020002176

**Published:** 2021-09

**Authors:** Thais S. Tavares, Fernanda L. B. Mügge, Viviane Grazielle-Silva, Bruna M. Valente, Wanessa M. Goes, Antonio E. R. Oliveira, Ashton T. Belew, Alessandra A. Guarneri, Fabiano S. Pais, Najib M. El-Sayed, Santuza M. R. Teixeira

**Affiliations:** 1 Departamento de Bioquímica e Imunologia, Universidade Federal de Minas Gerais, Belo Horizonte, 31270-901, Brazil; 2 Department of Cell Biology and Molecular Genetics and Center for Bioinformatics and Computational Biology, University of Maryland, 20742, College Park, Maryland, 20742, USA; 3 Center for Molecular Biology of Heidelberg University, Heidelberg, 69120, Germany; 4 Instituto René Rachou, Fundação Oswaldo Cruz, Belo Horizonte, MG, 30190-009, Brazil

**Keywords:** Parasite differentiation, post-transcriptional control, RNA binding proteins, transcriptome analyses, *Trypanosoma cruzi*

## Abstract

*Trypanosoma cruzi* has three biochemically and morphologically distinct developmental stages that are programmed to rapidly respond to environmental changes the parasite faces during its life cycle. Unlike other eukaryotes, Trypanosomatid genomes contain protein coding genes that are transcribed into polycistronic pre-mRNAs and have their expression controlled by post-transcriptional mechanisms. Transcriptome analyses comparing three stages of the *T. cruzi* life cycle revealed changes in gene expression that reflect the parasite adaptation to distinct environments. Several genes encoding RNA binding proteins (RBPs), known to act as key post-transcriptional regulatory factors, were also differentially expressed. We characterized one *T. cruzi* RBP, named TcZH3H12, which contains a zinc finger domain and is up-regulated in epimastigotes compared to trypomastigotes and amastigotes. TcZC3H12 knockout (KO) epimastigotes showed decreased growth rates and increased capacity to differentiate into metacyclic trypomastigotes. Transcriptome analyses comparing wild type and TcZC3H12 KOs revealed a TcZC3H12-dependent expression of epimastigote-specific genes such as genes encoding amino acid transporters and proteins associated with differentiation (PADs). RNA immunoprecipitation assays showed that transcripts from the PAD family interact with TcZC3H12. Taken together, these findings suggest that TcZC3H12 positively regulates the expression of genes involved in epimastigote proliferation and also acts as a negative regulator of metacyclogenesis.

## Introduction

*Trypanosoma cruzi*, the causative agent of Chagas disease, affects approximately 6–8 million people worldwide. It is endemic in Latin America, where it is a major public health problem and causes over 10 000 deaths annually (WHO, 2020). There is no vaccine and the two drugs currently available, in addition to having unwanted side-effects, are only effective in the acute phase of the infection (DNDi, 2020). Natural *T. cruzi* transmission to humans occurs from domiciliated triatomine insect vectors, mainly *Triatoma infestans* and *Rhodnius prolixus*. Although recent vector control programmes have succeeded in reducing infection rates in endemic areas, non-vectorial transmission routes, including transmission *via* contaminated food, blood transfusions, organ donation and congenital transmission, frequently occurs (WHO, 2020). When taking a blood meal on an infected mammal, the insect vector ingests circulating trypomastigotes, which, once inside the insect midgut, differentiate into replicative epimastigotes. In the posterior end of the digestive tract, epimastigotes differentiate into infective, non-dividing metacyclic trypomastigotes, which are eliminated with the insect's urine and feces during a blood meal. If inoculated through ruptures in the skin of a new mammalian host, trypomastigotes can enter the bloodstream and infect different cell types. Once inside the host cell, trypomastigotes differentiate into amastigotes that replicate in the cytoplasm for 3 to 5 days before differentiating again into highly motile trypomastigotes. Trypomastigotes cause lysis of the infected cells, reach the circulatory system and then propagate the infection by entering new cells or being ingested by a vector (Brener, 1973).

The existence of various morphologically and biochemically distinct life cycle stages that alternate between invertebrate and vertebrate hosts requires a finely regulated developmental programme. Similar to other members of the Trypanosomatid family, *T. cruzi* protein coding genes are organized into long polycistronic transcription units that are transcribed into polycistronic pre-mRNAs, which are subsequently processed into mature, monocistronic mRNAs through coupled trans-splicing and poly-adenylation reactions (El-Sayed *et al*., 2005). Because of polycistronic transcription, control of gene expression in *T. cruzi* must rely on post-transcriptional mechanisms, which occur mainly through a fine tuning of steady state levels and translation efficiency of the mRNA populations (Araújo and Teixeira, 2011; Clayton, 2019). Through direct or indirect RNA–protein interactions, RNA Binding Proteins (RBPs) are key elements that regulate the expression Trypanosomatid genes (Clayton, 2019). A large variety of these proteins are encoded in the *T. cruzi* genome, the majority of those having RNA recognition motifs (RRMs) (De Gaudenzi *et al*., 2005), but also zinc finger domains (Kramer *et al*., 2010), Pumilio (Caro *et al*., 2006), among others. Studies on several RBPs have shown that they can bind to regulatory sequences mainly present in the 3′-untranslated regions (UTR) of mRNAs and associate with additional cellular machinery to control target mRNA degradation (Dallagiovanna *et al*., 2005, 2008; Pérez-Díaz *et al*., 2012, 2013) or to promote mRNA stabilization (Noé *et al*., 2008; Sabalette *et al*., 2019).

Previous *in silico* analysis have shown that the *T. cruzi* genome encodes approximately 50 different proteins with CCCH motif and studies with *Trypanosoma brucei* as well as with different *Leishmania* species have demonstrated that these proteins can exert important functions in controlling stage-specific gene expression and parasite growth and differentiation (Kramer *et al*., 2010). Two small proteins named TcZFP1 and TcZFP2, whose homologues in *T. brucei* have been implicated in regulating parasite differentiation (Hendriks *et al*., 2001), have also been characterized in *T. cruzi* as zinc finger RBPs that interact with each other and are differentially expressed throughout the life cycle (Caro *et al*., 2005). Although expression of TcZFP1 is increased in metacyclic trypomastigotes (Espinosa *et al*., 2003; Mörking *et al*., 2004), TcZFP2 levels are reduced in this stage (Mörking *et al*., 2012). In contrast, the zinc finger protein TcZC3H39 is expressed constitutively among all life cycle stages but is differentially associated with distinct mRNA targets in response to nutritional stress (Alves *et al*., 2014). As the only *T. cruzi* RBP that has been functionally characterized using reverse genetics, TcZC3H31 was identified as a positive regulator of metacyclogenesis. TcZC3H31 gene knockout (KO) resulted in inhibited metacyclogenesis and an arrest of epimastigotes into an intermediate state (Alcantara *et al*., 2018). It has been shown that differentiation between life cycle stages that occurs into the insect and mammalian hosts is accompanied by changes in the expression of a large number of *T. cruzi* genes (Smircich *et al*., 2015; Li *et al*., 2016; Cruz-Saavedra *et al*., 2020*a*, 2020*b*). By performing global gene expression analysis, we have identified all RBPs with differential expression (DE) between epimastigotes, trypomastigotes and amastigotes. The gene TcCLB.506739.99, which encodes a zinc finger protein with orthologues in other Trypanosomatids (Ouna *et al*., 2012) but not yet characterized in *T. cruzi*, showed a 10-fold higher expression in epimastigotes when compared to the mammalian stages. The increased expression in epimastigotes suggests that this RBP may be involved with gene expression control during *T. cruzi* differentiation. Characterization of KO cell lines for this zinc finger protein provided evidence for its role in controlling mRNA levels of epimastigote-specific genes and suggests that it may act as a positive regulator of epimastigote growth and a negative regulator of metacyclogenesis.

## Materials and methods

### Parasite cultures

Epimastigote forms of *T. cruzi* CL Brener clone were maintained at 28°C in liver infusion tryptose (LIT) medium supplemented with 10% of fetal bovine serum (Gibco, USA) and penicillin/streptomycin (Gibco, USA) as previously described (CAMARGO, 1964). Cultures were maintained in the exponential growth phase by doing two to three dilutions per week in fresh LIT medium.

### DE analyses of total *T. cruzi* genes

Differential gene expression analyses were performed between epimastigote samples and tissue cultured trypomastigotes or intracellular amastigote samples from the RNA-Seq data published by Belew *et al*. (2017). For a description of samples, accession numbers and metrics, see Table S1. A total of 22 014 genes passed the low counts filter (applied to the raw counts of all 25 099 genes deposited in CL Brener genome v.38) and the DE analysis was performed using the DESeq2 package (Love *et al*., 2014). Genes were considered differently expressed (DEG) when they presented an adjusted *P* value (*P*_adj_) <0.05 and an absolute value of fold change in log base 2 (|log 2 fold change|) ⩾1. For gene ontology (GO) enrichment analysis the up-regulated DEG in epimastigote samples were analysed using the goseq package (Young *et al*., 2010) in two approaches, including and excluding genes related to the six largest gene families in the *T. cruzi* genome (‘trans-sialidase’, ‘MASP’, ‘mucin’, ‘RHS’, ‘DGF-1’, ‘GP63’). The terms presenting an overrepresented *P* value < 0.05 were considered enriched.

### *In silico* analyses of *T. cruzi* RBPs

To obtain the genes encoding for RBPs in the *T. cruzi* genome, Pfam and Interpro databases were used to obtain sequences that determine RNA-binding domains [RRM, PABP, PUF, Alba KH (types I and II), zinc finger (CCHC and CCCH), S1, PAZ, PIWI, TRAP and SAM]. Tool ‘Protein features and properties – Interpro Domain’ from the TritrypDB database (release 47) was used to identify the genes encoding RBPs based on the presence of the domain of interest in the analysed gene sequence. All *T. cruzi* genes containing RNA-binding domains identified in Table S2 were analysed in the three main life cycle stages of this parasite using data from DE analysis. Amino acid sequences corresponding to orthologues of RBPs in different Trypanosomatids were extracted from the TritrypDB database and used to perform multiple sequence alignments using T-Coffee (http://tcoffee.crg.cat/apps/tcoffee/do:mcoffee). Phylogenetic analysis was built using the method ‘neighbour joining’ in the CIPRES Science Gateway (https://www.phylo.org/) and edited by iTOL – interactive tree of life (https://itol.embl.de/).

### Expression of HA-tagged TcZC3H12

Genomic DNA from CL Brener strain was extracted using the Illustra blood genomic Prep Mini Spin Kit (GE Healthcare, USA). A total of 200 ng of DNA were used to amplify the complete coding sequence of TcCLB.506739.99 by polymerase chain reaction (PCR), using forward For_TcZC3H12_XbaI and reverse Rev_TcZC3H12-HA_XhoI primers containing XbaI and XhoI restriction sites, respectively (Table S3). Reverse primer also contains the sequence corresponding to HA epitope, to be inserted in the C-terminal region of the TcZC3H12 sequence. Amplicons were purified and inserted in pROCKGFPNeo (DaRocha *et al*., 2004) expressing vector, previously digested with XbaI and XhoI, generating pROCK.ZC3H12-HA-Neo plasmid. This plasmid was digested with XbaI and NheI enzymes to release TcZC3H12-HA sequence followed by the neomycin expression cassette and treated with one Taq DNA polymerase (NEB) to allow for cloning in pCR2.1-TOPO plasmid (Invitrogen). Each construct was confirmed by PCR using specific primers. For transfection, (1) 5 *μ*g of TOPO-TcZC3H12-HA were digested with AflII; and (2) 20 *μ*g of pROCK.ZC3H12-HA-Neo, were digested with NotI. In each case, 2 × 10^7^ epimastigotes were resuspended in 100 *μ*L of Tb-BSF buffer (Schumann Burkard *et al*., 2011) and subjected to the programme U033 in AMAXA Nucleofector (Lonza). Twenty-four hours after transfection, parasites were selected with G418 (200 *μ*g mL^−1^) antibiotic. For pROCK.ZC3H12-HA-Neo, clones were obtained after serial dilutions to 0.5 parasites per well. Expression of TcZC3H12 with HA tag was confirmed by using a western blot assay. Briefly, epimastigotes were lysed in 2× SDS loading buffer containing protease inhibitor cocktail (Merck), and a volume corresponding to 2 × 10^6^ cells *μ*L^−1^ was loaded into 12% SDS-polyacrylamide gel. After transferring to nitrocellulose membrane, incubation was done with 1:2000 primary anti-HA (Sigma) and 1:3000 secondary HRP anti-immunoglobulin (IgG) (Sigma) antibodies.

### Fluorescence microscopy

For cellular localization, log-phase cultures or cultures aged in LIT medium from epimastigotes expressing endogenous TcZC3H12 with HA tag were centrifuged and washed with phosphate-buffered saline (PBS) and later fixed with 4% paraformaldehyde for 10 min. Following steps were performed with parasites in suspension and washing steps consisted of resuspension followed by centrifugation at 400 × ***g***. After washing with PBS, parasites were blocked and simultaneously permeabilized with blocking solution (PBS–1% BSA–0.2% Triton X-100) for 20 min at room temperature. Then, parasites were incubated with 1:250 anti-HA antibody (Millipore) in PBS–0.2% BSA overnight at 4°C. After washing with PBS–0.2% BSA, parasites were incubated with secondary anti-mouse IgG conjugated to Alexa Fluor 488 (Invitrogen, USA) for 30 min in the dark. After washing, 1 *μ*g mL^−1^ of diamidino-2-phenylindole (DAPI) (Molecular Probes/Life Technologies, USA) was used for nuclei staining. Parasites were resuspended in Prolong Gold anti-fade (Molecular Probes/Life Technologies, USA) and mounted in glass slides topped with glass coverslips, sealed with nail polish. Images were captured on a Zeiss LSM 980 Airyscan 2 laser scanning confocal microscope using a 60× oil immersion objective, located in the Imaging Core of the Department of Cell Biology and Molecular Genetics of University of Maryland. Image processing was performed in Zen Black software.

### Generation of TcZC3H12 KO cell lines

DNA constructs were generated to disrupt both TcZC3H12 alleles. The first allele was disrupted by homologous recombination. For this, PCR amplicons corresponding to 5′ (from 150 bp before the first ATG until 250 bp after that) and 3′ (from 200 bp before stop codon until 200 bp after stop codon) sequences of the gene were obtained. Additionally, restriction sites for HindIII/SacI and XhoI/XbaI enzymes were added in 5′ and 3′, respectively (for specific primer sequences see Table S3). PCR product corresponding to the 5′ was first cloned upstream of pTopo_HX1_Neo_GAPDH (Grazielle-Silva *et al*., 2015) and then the 3′ fragment was cloned downstream of GAPDH region, generating the plasmid TcZC3H12_Neo. To generate single KO parasites, this plasmid was used as the PCR template using the primers For5′KO_TcZC3H12_HindIII and Rev3′KO_TcZC3H12_XbaI. These PCR products were used to transfect wild type (WT) parasites. Twenty-four hours post transfection single KO parasites were selected with G418 (200 *μ*g mL^−1^) antibiotic. To delete the second allele, the plasmid TcZC3H12_Neo was digested with SpeI and NotI restriction enzymes and cloned into the plasmid Topo_HX1_Hygro_GAPDH, previously digested with the same enzymes. After that we obtained the plasmid TcZC3H12_Hygro. PCR products generated by amplification with primers For5′KO_TcZC3H12_HindIII and Rev3′KO_TcZC3H12_XbaI, were used as donor sequences to disrupt the second allele using sgRNAs (for specificities see Table S3) and recombinant Cas9 derived from *Staphylococcus aureus* (rSaCas9) as previously described by Soares Medeiros *et al*. (2017) and Burle-Caldas *et al*. (2018). After 24 h, hygromycin (200 *μ*g mL^−1^) was added to the medium already containing G418 for resistant parasites selection and clones were obtained by limiting dilution after platting 0.5 parasites per well. Addback parasites were generated from a KO clone transfected with pROCK.ZC3H12-HA plasmid linearized with NotI in which the neomycin resistance gene was replaced by the puromycin resistance sequence.

### *In vitro* metacyclogenesis

Metacyclogenesis was induced by starvation in aged LIT medium, as described by Shaw *et al*. (2016). For this, epimastigotes that were kept in exponential-growth phase were transferred to new culture flasks containing fresh LIT supplemented with fetal bovine serum and penicillin/streptomycin to a density of 2 × 10^6^ mL^−1^. The cell suspensions were left undisturbed for the parasites to attach to the bottom of the bottles for a period of 9 and 11 days. After these specific times, aliquots of each flask were collected, and parasites were fixed on glass slides and stained with Giemsa. Briefly, parasite smears were air dried, fixed with methanol 100% for 1 min and stained with 3% Giemsa stain (3% Giemsa stock in 0.95% m/v sodium phosphate dibasic, 0.9% m/v potassium phosphate monobasic, pH 7.0) for 30 min. After staining, the smears were washed in running water and air dried. This staining allows the identification of epimastigotes and metacyclic trypomastigotes based on the location of the kinetoplast in relation to the nucleus. Cells were counted on a light microscope using the 100× objective with immersion oil and the percentage of metacyclic trypomastigotes was calculated in relation to a total of 500 parasites per slide.

### Infection of *R. prolixus* bugs to assess *in vivo* metacyclogenesis

*Rhodnius prolixus* bugs used in this study were obtained from the Vector Behaviour and Pathogen Interaction Group from René Rachou Institute (IRR, Fiocruz, MG, Brazil). Triatomines were maintained under a controlled environment with a relative humidity of 26 ± 1°C, 50 ± 5% and natural illumination. WT or TcZC3H12 KO epimastigotes were added to citrated heat-inactivated (56°C/30 min) rabbit blood at a concentration of 1 × 10^7^ epimastigotes mL^−1^. Rabbit blood was provided by CECAL (Centro de Criação de Animais de Laboratório, Fiocruz, Rio de Janeiro). Fourth instar nymphs were fed with these parasite suspensions using an artificial feeding apparatus (Guarneri, 2020). Insects from each group that did not feed were removed from the containers. After 15 days bugs were expected to moult. After additional 20 days, bugs were fed again, this time on chickens anesthetized with intraperitoneal injections of ketamine (20 mg kg^−1^; Cristália, Brazil) and detomidine (0.3 mg kg^−1^; Syntec, Brazil). The use of chickens followed established procedures of Fiocruz and was approved by the Ethics Committee on Animal Use (CEUA-FIOCRUZ) under the license number LW-8/17. After feeding, each bug was transferred to a 1.5 mL microcentrifuge tube for urine collection. Smears were prepared from the urine and afterwards stained for parasite counting. The bugs were returned to containers and kept for 10 days more under the same conditions. After this period, the bugs were dissected and the intestine, separated into three portions (anterior midgut, posterior midgut and rectum), was macerated in 50 *μ*L of PBS and analysed under the microscope. As no parasites were found in the anterior midgut and forms in the posterior midgut were rarely observed, only those parasites of the rectum were counted. Urine smear staining was performed with Giemsa as previously described. Differential counting of epimastigotes and metacyclic trypomastigotes was performed under a 100× objective with immersion oil and the percentage of metacyclic trypomastigotes for each bug was calculated.

### RNA extraction and quantitative PCR analyses

Total RNA from epimastigote cultures was extracted using TRIzol reagent (Invitrogen) following the manufacturer's instructions. After DNase treatment (Invitrogen), first strand of cDNA was obtained from 200 ng of total RNA using SuperScript II Reverse Transcriptase (Invitrogen) and OligodT. Specific primers (Table S3) and SSO SYBR Green Supermix (Bio-Rad) were used in quantitative PCR (qPCR) reactions following the manufacturer's instructions. An Applied Biosystems 7900HT Fast Real-Time PCR System (Life Technologies) platform was used to run the reactions. Relative mRNA levels were normalized to *Ct* values for the gene encoding for constitutive 60S ribosomal protein L9 (TcCLB.504181.10), following the 2^−ΔΔ*ct*^ method (Livak and Schmittgen, 2001).

### RNA sequencing and bioinformatics analysis

Total RNA was extracted from 10^8^ WT or TcZC3H12 KO epimastigotes from exponentially growing cultures using the Illustra RNAspin Mini kit (GE Healthcare). cDNA synthesis was performed using SuperScript II Reverse Transcriptase kit first-strand synthesis (Invitrogen) and Oligo(dT)_18_, according to the manufacturer's instructions. The quality of RNA was determined by using an Agilent 2100 bioanalyzer and quantified by qPCR using a KAPA Biosystems library quantification kit. RNA coming from duplicates of two clones TcZC3H12 null-mutants and triplicates from WT *T. cruzi* CL Brener clone were subjected to the Illumina^®^ TruSeq^®^ RNA Sample Preparation Kit v2 to generate RNA-Seq libraries. Illumina next-generation sequencing was performed using the MiSeq platform. Sequence quality metrics were assessed using FastQC (Andrews, 2010). Trimmomatic (Bolger *et al*., 2014) was used to remove any remaining Illumina adapter sequences from reads and to trim bases off the start or the end of a read when the quality score fell below a threshold of 25. The remaining reads were aligned against the concatenated *Esmeraldo*, *non-Esmeraldo* and *Unassigned* haplotypes of *T. cruzi* using hisat2 (Kim *et al*., 2019). Reads from multigene families (trans-sialidase, mucin, MASP, RHS, GP63 and DGF) were removed from the list with the aim to avoid interference in downstream analyses. The hisat2-derived accepted hits were sorted and indexed *via* SAMtools (Li *et al*., 2009) and passed to htseq (Anders *et al*., 2015) to generate count tables. The resulting data were passed to DESeq2 (Love *et al*., 2014) and a *de-novo* basic analysis which served as a ‘negative control’ for the statistical models employed. Pairwise contrasts were performed using the experimental condition and batch, or after applying sva (Leek *et al*., 2012) to the data. The pairwise results were compared across methods, and the interesting contrasts were extracted. Genes with DE were defined as genes with log 2 fold change >1 or log 2 fold change <1 and *P*_adj_ value < 0.05, between WT and null mutants.

### RNA immunoprecipitation

WT and endogenous TcZC3H12-HA expressing epimastigotes were incubated with 10 *μ*g mL^−1^ proteasome inhibitor (MG-132 – Calbiochem) for 1 h at 28°C. Following the incubation period, cells were centrifuged 3000 × ***g*** for 10 min at 4°C, resuspended in PBS and UV cross-linked two times at 240 mJ cm^−2^ on ice (UV Crosslinker, Spectroline). After centrifugation at 3000 × ***g*** for 10 min at 4°C and supernatant removal, cell pellets were snap frozen in liquid nitrogen. Pellets were then resuspended in RIPA buffer (Tris-HCl pH 8.0 5 mm; NaCl 15 mm; NP-40 0.1%, 0.05% sodium deoxycholate, 0.01% SDS) and incubated at 4°C for 10 min. Shearing was performed 10 times using 21G needle and five times using a 30G syringe. Lysates were centrifuged 8200 × ***g*** for 10 min and the supernatant transferred to a new tube. In total, 10% of cell lysate (input fraction) were transferred to a new tube and saved for further analysis. Remaining lysate was incubated with EZview Red Anti-HA Affinity Gel (Sigma-Aldrich) for 2 h at 4°C under soft agitation. Beads were then centrifuged 8000 × ***g*** for 5 min and unbound proteins, present in the supernatant, transferred to new tube. Beads were washed three times with lysis buffer and divided into two tubes: (1) for protein elution and (2) for RNA extraction. (1) Bound proteins were eluted in elution buffer (62.5 mm Tris-HCl pH 6.8; 10% glycerol; 2% SDS; 5% *β*-mercaptoethanol; 0.002% bromophenol blue). (2) Beads were resuspended in 1% SDS and treated with proteinase K (Invitrogen) for 30 min at 37°C. After proteinase K inactivation, RNA extraction was performed using TRIzol reagent. The same was performed for input fractions, following the manufacturer's instructions. The SuperScript IV One-step RT-PCR kit (ThermoFisher) was used for detection of transcripts, using specific primers for proteins associated with differentiation (PADs) and GAPDH (Table S3), following specifications. After RT-PCR, products were run on 0.5% agarose gel (Sigma), visualized and captured in an iBright equipment (ThermoFisher). Densitometry analysis was performed using ImageJ.

### Statistical analysis

Two-tailed *t*-test was performed for experiments in which variables had a normal distribution. Each experiment had at least three biological replicates. For variables that did not show a normal distribution Mann–Whitney non-parametric test was used. For all tests, the accepted significance level was *P* < 0.05.

### Data availability

RNA-Seq data were deposited at the National Center for Biotechnology (NCBI) Sequence Read Archive (SRA) under bioproject PRJNA389926 [for CL Brener strain WT epimastigotes, trypomastigotes and amastigotes – (Belew *et al*., 2017)] and bioproject PRJNA660030 (for comparative transcriptome of WT and TcZC3H12 KO epimastigotes).

## Results

### Identification of RBP genes up-regulated in epimastigotes

Previous transcriptome profiling analyses comparing trypomastigotes and intracellular amastigotes from two *T. cruzi* strains with distinct virulence phenotypes revealed essential factors involved in the parasite's adaptation to the mammalian host (Belew *et al*., 2017). Here, we extended our RNA-Seq data analyses to include the epimastigote stage (Table S1), and to compare the transcriptome profiles obtained from tissue culture-derived trypomastigotes and intracellular amastigotes obtained 60 h post-infection (hpi) with the transcriptome obtained from *in vitro* cultured epimastigotes of the CL Brener cloned strain, which has not been reported before. Mapped-sequencing data derived from the epimastigote samples were analysed using principal component analysis (PCA) to inspect relationships between samples (Fig. S1). The resulting PCA plot after normalization showed the expected clustering between biological replicates. DE analyses (Table S4) revealed a total of 2489 transcripts with increased expression levels in epimastigotes (log 2 fold change >1, *P*_adj_ value < 0.05) and 624 transcripts with reduced expression levels compared to amastigotes. Using similar criteria to identify statistically significant DE genes, we compared the epimastigote and trypomastigote global transcriptomes. A total of 2404 transcripts were identified as significantly up-regulated in epimastigotes whereas 3525 transcripts showed reduced expression levels. Among a total of 3780 transcripts that showed increased levels in epimastigotes compared to the other two forms (log 2 fold change >1 and *P*_adj_ value < 0.05), GO analyses revealed groups of genes encoding proteins involved with oxidation-reduction process (GO:0055114), ion transport (GO:0006811), amino acid metabolism (GO:0006520), translation (GO:0006520) and ATP metabolic process (GO:0046034). These results are in accordance with previously published epimastigote transcriptomes from other *T. cruzi* strains, namely Dm28c, Y and Sylvio (Smircich *et al*., 2015; Li *et al*., 2016; Cruz-Saavedra *et al*., 2020*b*). Also, members of the beta amastin sub-family, whose epimastigote-specific expressions have been previously described by our group (Kangussu-Marcolino *et al*., 2013), as well as members of the family of PADs, which have been characterized in *T. brucei* (Dean *et al*., 2009) but not in *T. cruzi*, were also found among the genes that are up-regulated in CL Brener epimastigotes (Table S5).

**Fig. 1.**
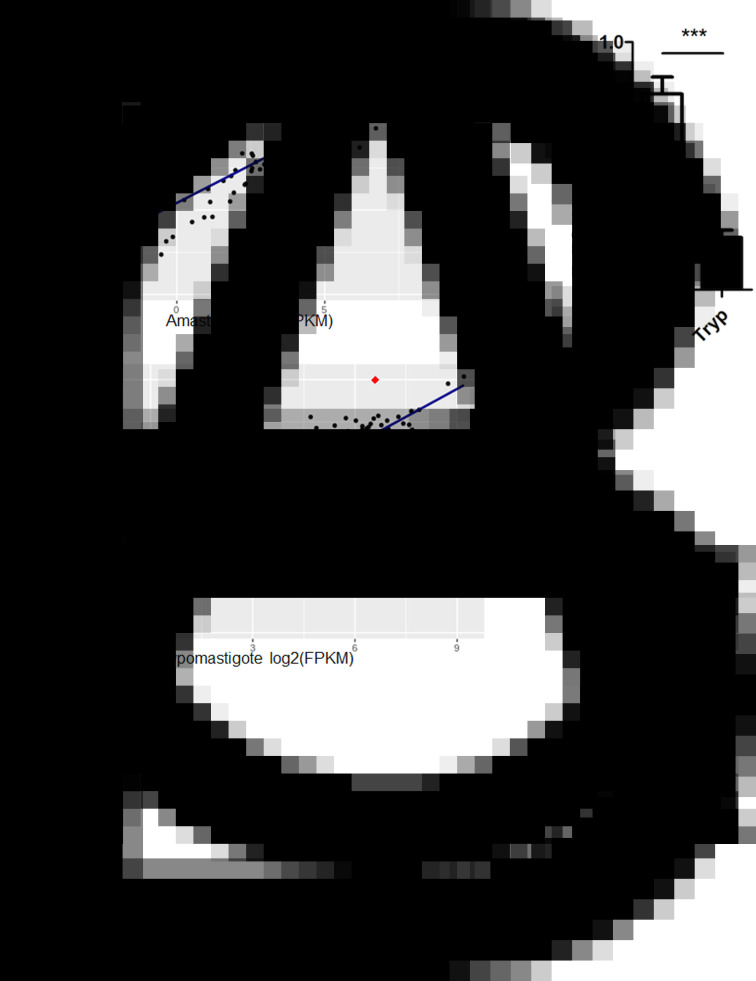
TcZC3H12 is an RBP that is up-regulated in epimastigotes. RNA-Seq data published by Belew *et al.* (2017) were used to identify 175 *Trypanosoma cruzi* genes coding for RBPs. (A) Scatterplot comparing mRNA levels of RBPs in epimastigote and amastigote or (B) epimastigote and trypomastigote. Each black dot corresponds to a different RBP transcript. Red diamond in each plot corresponds to TcZC3H12 (TcCLB.506739.99). (C) Real-time PCR data showing that TcZC3H12 are up-regulated in epimastigotes compared with tissue culture-derived trypomastigotes obtained after infection of LLC-MK2 cell monolayers (*n* = 3; ***P < 0.001).

**Fig. 2.**
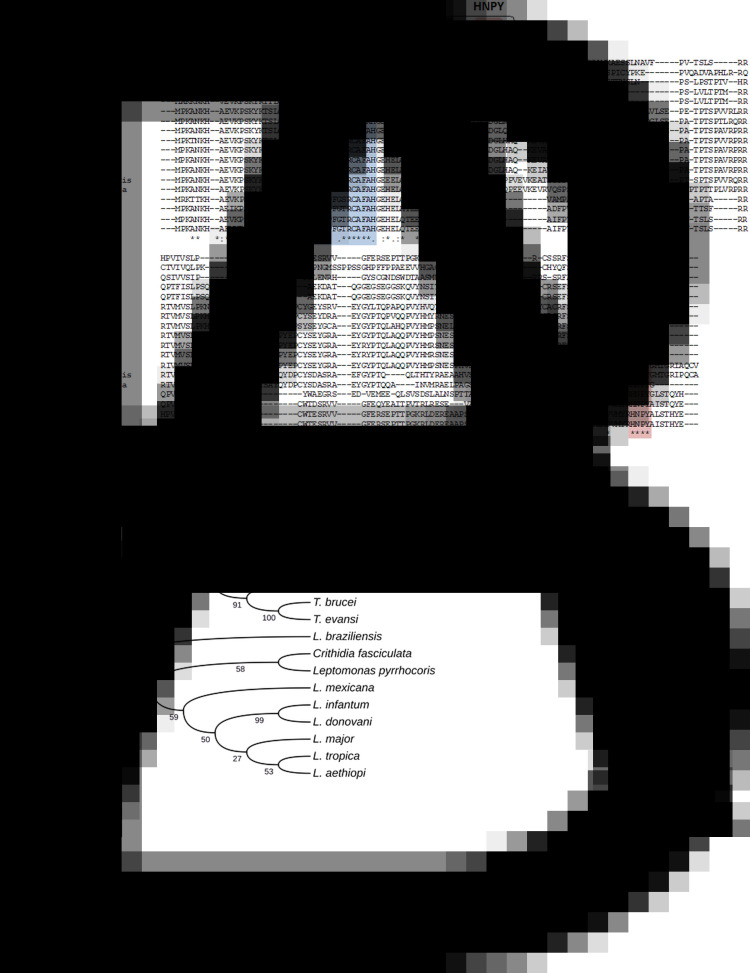
TcZC3H12 orthologues are conserved in kinetoplastids. (A) Schematic representation of predicted relevant domains of TcZC3H12. Orthologous sequences were extracted from TriTrypDB data bank and used to generate a multiple sequence alignment and (B) a phylogenetic tree. Some orthologues were represented by the abbreviated genus name, where ‘L.’ is *Leishmania* and ‘T.’ is *Trypanosoma*. TritrypDB identifiers used were: TcCLB.506739.99 (*T. cruzi* Esmeraldo), TvY486_0501060 (*T. vivax*), TevSTIB805.5.1760 (*T. evansi*), Tb927.5.1570 (*T. brucei*), LbrM.15.0140 (*Leishmania braziliensis*), LmxM.15.0140 (*L. mexicana*), LINF_150006300 (*L. infantum*), LdCL_150006300 (*L. danovani*), LMJLV39_150006500 (*L. major*), CFAC1_060021400 (*Crithidia fasciculata*), DQ04_00711020 (*Tropidophorus grayi*), TRSC58_03397 (*T. rangeli*), LpyrH10_38_0260 (*L. pyrrhocoris*), C4B63_83g24 (*T. cruzi* Dm28), C3747_176g18 (*T. cruzi* TCC), LAEL147_000208800 (*L. aethiopica*), LTRL590_150006300 (*L. tropica*). Values next to the branching points indicate the relative support from 100 bootstrap replicates.

Because post-transcriptional mechanisms mediated by RBPs play a major role in controlling gene expression in *T. cruzi*, we searched for RBPs that may act as regulators of epimastigote genes by comparing transcript levels of all RBP genes between epimastigotes and the two other forms of the parasite. Although 253 *T. cruzi* sequences encoding RBPs were previously identified in the CL Brener genome (Belew *et al*., 2017), homology searches based on protein domains that included additional motifs known to be present in RBPs resulted in the identification of 297 sequences that correspond to 175 RBP genes (Table S2). As indicated in our previous study, because the CL Brener strain has a hybrid genome (El-Sayed *et al*., 2005), for most genes, we identified two distinct alleles, corresponding to the Esmeraldo and the non-Esmeraldo haplotypes. DE analysis identified over 20 RBPs that are developmentally regulated in *T. cruzi*, the majority of them being identified when epimastigote and trypomastigote stages were compared (Table S6). Among all RBP genes whose expression is up-regulated in epimastigotes, we identified the gene annotated as TcCLB.506739.99 as the one with the largest difference in expression when epimastigotes were compared to trypomastigotes. Based on the RNA-Seq data, transcript levels for the TcCLB.506739.99 gene presented a 10-fold increase in epimastigotes compared to trypomastigotes and a 7-fold increase compared to amastigotes (Fig. 1A and B, respectively). Differences in transcript levels found for the non-esmeraldo allele for the same gene (TcCLB.510819.119) were 4-fold (comparing epimastigotes to trypomastigotes) and 3-fold (comparing epimastigotes to amastigotes). Quantitative PCR analysis performed with RNA obtained from cultured epimastigotes and tissue culture-derived trypomastigotes confirmed that the TcCLB.506739.99 gene is up-regulated in the insect stage of the parasite (Fig. 1C). These results are in accordance with previous RNA-Seq studies that showed higher expression of this gene in two other *T. cruzi* strains, namely Y strain (Li *et al*., 2016), and Dm28c strain (Smircich *et al*., 2015). In TriTrypDB (http://www.tritrypdb.org), TcCLB.506739.99 is annotated as a gene encoding a 19.5 kDa RBP that contains a zinc finger motif and presenting orthologues in the genomes of several other members of Trypanosomatid family including *T. brucei*. Interestingly, no similar sequences were found in the genomes of other eukaryotes. The *T. brucei* orthologue, Tb927.5.1570, encodes TbZC3H12, an RBP that is up-regulated in the procyclic forms of the parasite, the stage found in the tsetse fly (Ouna *et al*., 2012; Fernández-Moya *et al*., 2014). Multiple sequence alignments revealed a high degree of conservation between orthologues from several Trypanosomatids at the N-terminal region (Fig. 2A), where the zinc finger domain is localized (C-X8-C-X5-C-X3-H), as well as in the C-terminal region where a HNPY motif is located. In *T. brucei*, the HNPY motif has been characterized as a MKT1-PBP1 binding motif (Singh *et al*., 2014) involved in the formation of a translation regulatory complex (Tadauchi *et al*., 2004). As shown in Fig. 2B, a phylogenetic tree built with amino acid sequences derived from various members of the Trypanosomatid family showed that TcZC3H12 homologues can be clustered in two sub-families: a sub-family comprising *Trypanosoma* sequences and a sub-family that includes sequences derived from *Leishmania*, *Leptomonas* and *Crithidia* spp.

### TcZC3H12 has a cytoplasmic localization and decreased protein levels after metacyclogenesis

**Fig. 3.**
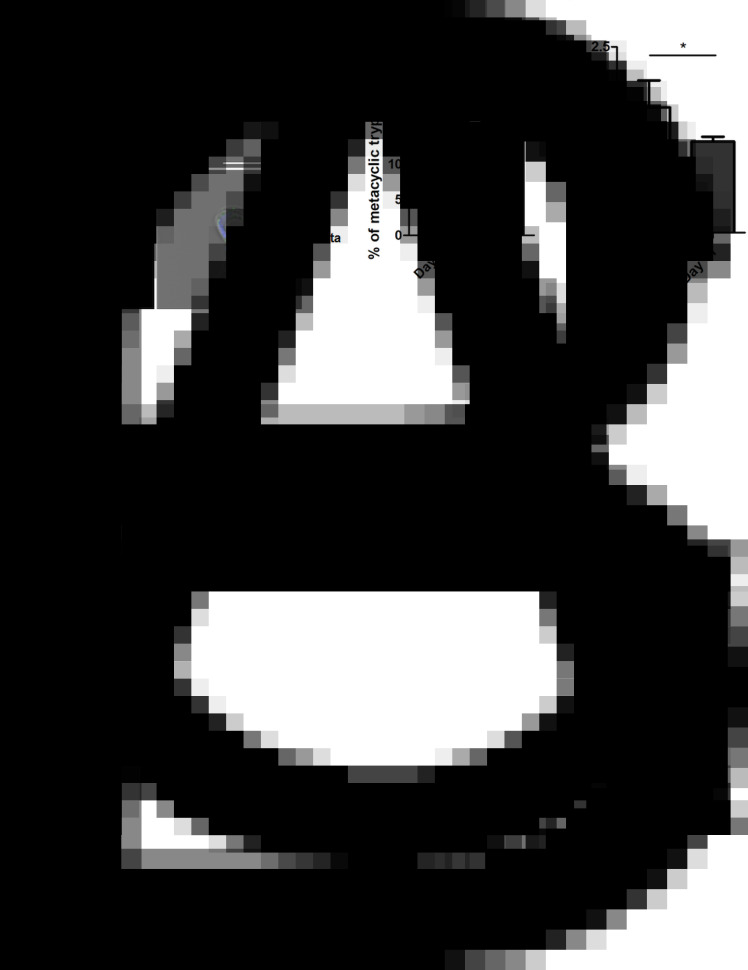
Cellular localization and expression levels of TcZC3H12 in log phase and stationary phase epimastigote cultures. (A) Epimastigotes expressing TcZC3H12 fused to an HA epitope were diluted every 2 days in LIT medium to be kept in the log phase. The parasites were fixed in 4% paraformaldehyde and incubated with primary mouse anti-HA antibody and secondary anti-mouse antibody conjugated to Alexa-488 (top panels). To induce metacyclogenesis, epimastigotes were kept in LIT medium without changing medium for 9 days and parasites were fixed and stained using the same protocol (bottom panels). Nuclei and kinetoplast were stained with DAPI, allowing the identification of metacyclic trypomastigotes. (B) The numbers of metacyclic trypomastigotes were determined on days 5 and 11 of culture in LIT medium after Giemsa staining (**P < 0.01; *n* = 3). (C) Real-time PCR showing the relative abundances of TcZC3H12 mRNA in epimastigote obtained from log phase and stationary phase cultures. Total RNA was extracted from cultures growing in LIT medium for 5 or 77 days and RT-qPCR was performed with TcZC3H12-specific primers and primers for the TcRPL9 gene as internal control (*P < 0.05; *n* = 2).

To investigate the role of TcZC3H12 in *T. cruzi*, we generated epimastigote cell lines expressing an HA-tagged version of this protein. The coding region of TcCLB.506739.99 was amplified from DNA extracted from the CL Brener clone using a set of primers to add a C-terminal HA tag. The PCR product was used to transfect CL Brener epimastigotes to allow homologous recombination with the endogenous gene. Using an anti-HA antibody, we determined the cellular localization of the protein by performing immunofluorescence assays of log phase and stationary phase epimastigotes cultivated in LIT medium. As described by (Figueiredo *et al*., 2000), parasites that were maintained in LIT medium for more than seven days without changing medium, i.e. in the stationary phase of growth, were subjected to a culture condition that induces epimastigote differentiation into metacyclic trypomastigotes due to nutritional starvation. As shown in the top panels of Fig. 3A, TcZC3H12 has a cytoplasmic localization in the log phase, replicative epimastigotes, where it accumulates in several foci mainly located in the posterior portion of the parasite body. In metacyclic trypomastigotes (bottom panels), present in the stationary phase (day 11, Fig. 3B), a much weaker fluorescence signal was observed. We quantified the transcript levels of TcZC3H12 in the log phase and stationary phase parasite cultures, by performing RT-qPCR with RNA extracted from day 5 of culture, during which no metacyclic trypomastigotes are present and day 11, during which approximately 15% of the parasites have the morphology of metacyclic trypomastigotes (Fig. 3B). In agreement with the immunofluorescence data, Fig. 3C shows a 25% decrease in TcZC3H12 mRNA levels obtained from cultures containing metacyclic trypomastigotes when compared to cultures containing only log phase epimastigotes, thus indicating that TcZC3H12 expression is down-regulated during epimastigote-metacyclic trypomastigote differentiation.

### TcZC3H12 KO impairs epimastigote growth and increases metacyclogenesis

**Fig. 4.**
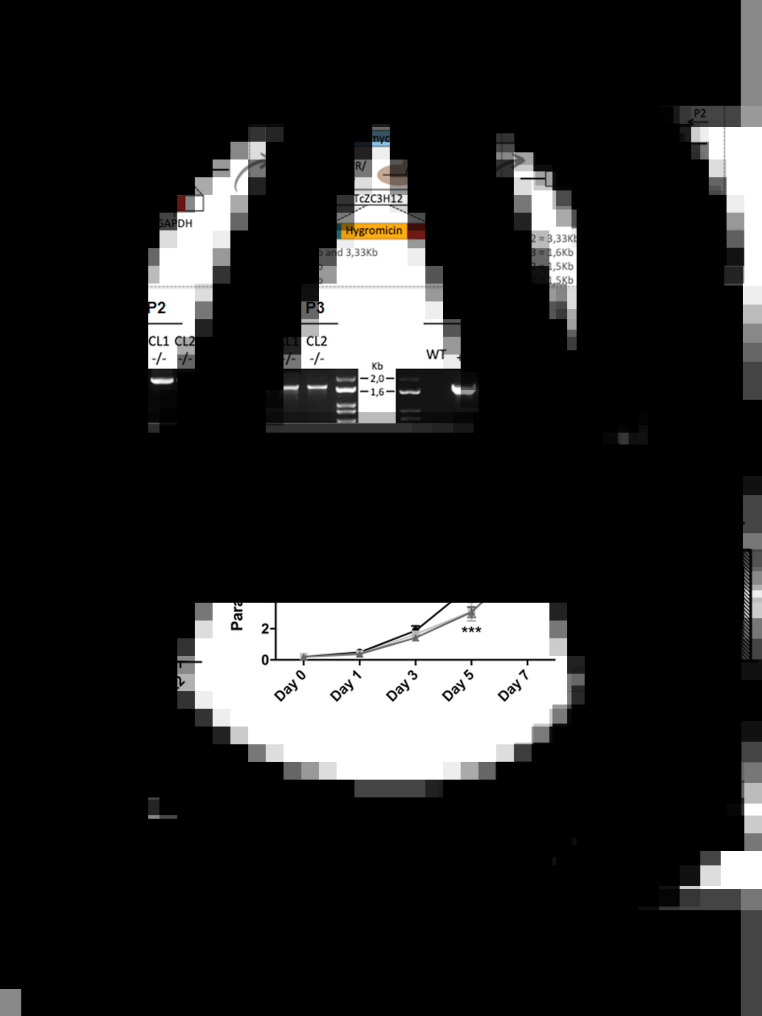
TcZC3H12 KO parasites have altered growth rates and metacyclogenesis *in vitro*. (A) Schematic representation of the generation of KO cell lines. (B) Agarose gel showing PCR products from different primer combinations to verify the correct integration of the DNA constructs. P1 + P2 in WT parasites amplified the TcZC3H12 coding region and part of the UTR region (1274 pb). In the KO cell lines the primers align outside to the inserted constructs and amplified their sequence plus some nucleotides of the UTR region (3334 bp – interruption by neomycin sequence and 3382 bp – interruption by hygromycin sequence). Neomycin resistance gene integration in TcZC3H12 locus was verified by amplification with primers P1 + P3 (1653 bp) and P2 + P4 (1582 bp). P5 + P2 (1586 bp) amplified hygromycin resistance gene integrated in the TcZC3H12 locus. (C) Relative expression levels of TcZC3H12 quantified by RT-qPCR using RNA extracted from WT and two KO cell lines (KO.1 and KO.2). (D) Growth curves and (E) *in vitro* metacyclogenesis assays to compare the percent of metacyclic trypomastigotes of WT, KO.1 and KO.2 parasites [*n* = 3 for (C), (D) and (E); ***P < 0.001 and **P < 0.01].

To directly assess the role of TcZC3H12, we generated KO cell lines by disrupting both alleles through homologous recombination with neomycin-resistance and hygromycin-resistance cassettes followed by selection in LIT medium containing G418 and hygromycin. To improve the efficiency of the process, the second allele was disrupted using the CRISPR/Cas9 technology by transfecting parasites with recombinant Cas9 and a small guide RNA targeting this allele (this strategy is illustrated in Fig. 4A). DNA extracted from two clones, selected from the G418 and hygromycin resistant population, was tested by PCR using different combinations of primers. The results of PCR amplification using primers P1 and P3 (generating a 1653 bp product) as well as P2 and P4 (generating a 1582 bp product) confirmed the correct insertion of the neomycin resistance cassette into the first allele, generating a heterozygous mutant. PCR products using primers P5 and P2 (generating a 1586 bp product) confirmed the insertion of the hygromycin resistance cassette into the second allele. As expected, PCR products generated with primers P1 and P2 amplified a fragment corresponding to the TcZC3H12 coding region and part of the UTR region (1274 bp) only with DNA extracted from WT parasites but not with DNA from clones 1 and 2, confirming the disruption of both alleles by the drug resistance cassettes (Fig. 4B). Furthermore, as shown in Fig. 4C, qPCR using total RNA and primers targeting the TcZC3H12 mRNA resulted in PCR products only with RNA from WT parasites but no PCR products were obtained with RNA from both KO cell lines. To test the effect of TcZC3H12 disruption on epimastigote growth, we inoculated the two KO cell lines as well as WT epimastigotes in LIT medium and determined the cell densities for up to 7 days. As shown in Fig. 4D, the KO parasites presented lower growth rates compared to WT, with a difference that is highly evident on day 7. This result indicates that expression of TcZC3H12 is required for epimastigote proliferation and its absence may provide a signal for the parasite to enter the stationary phase. As indicated before, metacyclogenesis can be reproduced *in vitro* under starving conditions that can be mimicked by culturing epimastigotes for more than 7 days in LIT medium (Figueiredo *et al*., 2000). Comparing to WT epimastigotes, which, upon entering the stationary phase of the growth curve, have about 15% of its parasite population differentiated into metacyclic trypomastigotes, the two TcZC3H12 KO cell lines presented a percentage of metacyclic trypomastigotes that is 20–30% higher after 9 days in culture (Fig. 4E). Such differences in the growth and metacyclogenesis rates between WT parasites and TcZC3H12 KO mutants indicate that the TcZC3H12 RBP may act a regulatory factor that controls genes involved with epimastigote proliferation and differentiation in the insect vector.

**Fig. 5.**
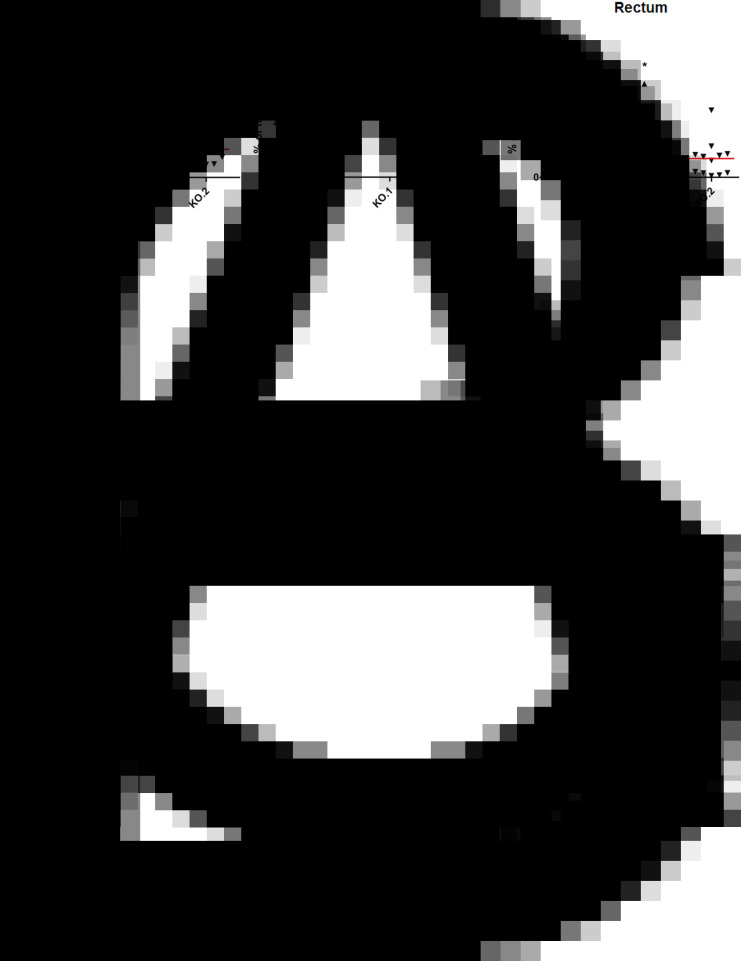
Increased numbers of metacyclic trypomastigotes in the excreta of triatomines infected with TcZC3H12 KO parasites. *Rhodnius prolixus* bugs were infected with WT and two KO cell lines (KO.1 and KO.2). Each point represents urine or rectum samples from different infected insects. Median values are displayed as red lines. (A) Total parasite numbers (epimastigotes + metacyclic trypomastigotes) inside the rectum were counted after maceration in PBS and without fixation (WT: *n* = 15; KO.1: *n* = 18; KO.2: *n* = 12). Percentage of metacyclic trypomastigotes found in samples of (B) urine (WT: *n* = 6; KO.1: *n* = 7; KO.2: *n* = 6) and (C) rectum (WT: *n* = 23; KO.1: *n* = 19; KO.2: *n* = 24) were calculated after sample fixation and Giemsa staining (number of metacyclic trypomastigotes/number of total parasites) (*P < 0.05 in Mann–Whitney's test).

To verify whether the differences in growth and differentiation between WT and TcZC3H12 KO parasites also occur *in vivo*, *R. prolixus* insects were infected with two KO cell lines or with WT epimastigotes. Fourth instar nymphs were fed with citrated heat-inactivated blood containing epimastigotes from each cell line. As previously described, parasites from CL strain migrate and colonize the insect digestive tract, differentiating into metacyclic trypomastigotes within 30–45 days (Ferreira *et al*., 2016). A second blood meal was offered 30 days after the infection and the urine eliminated by the insects immediately after the feeding was collected. Ten days after the second feeding, the insects were dissected, and the rectum was analysed under the microscope. Total parasite numbers and the percentage of metacyclic trypomastigotes were determined in the urine and rectum samples. As shown in Fig. 5A, no significant differences in total parasite numbers obtained from the digestive tract were observed, although a tendency towards higher numbers of WT parasites compared to KO cell lines can be noted. In urine samples, a natural source of infections by *T. cruzi*, the percentages of infectious metacyclic trypomastigotes were about 2-fold higher in insects infected with KO cell lines compared to insects infected with WT parasites (Fig. 5B). Although less evident compared to the results observed in urine samples, the percentage of metacyclic trypomastigotes found in the rectum was also higher in insects infected with KO parasites than with WT (Fig. 5C). Thus, the presence of higher proportion of metacyclic trypomastigotes in the rectum and in the urine of infected triatomines corroborates the results obtained with *in vitro* experiments which showed that, in the absence of the TcZC3H12 gene, *T. cruzi* epimastigotes undergo metacyclogenesis at higher rates.

**Fig. 6.**
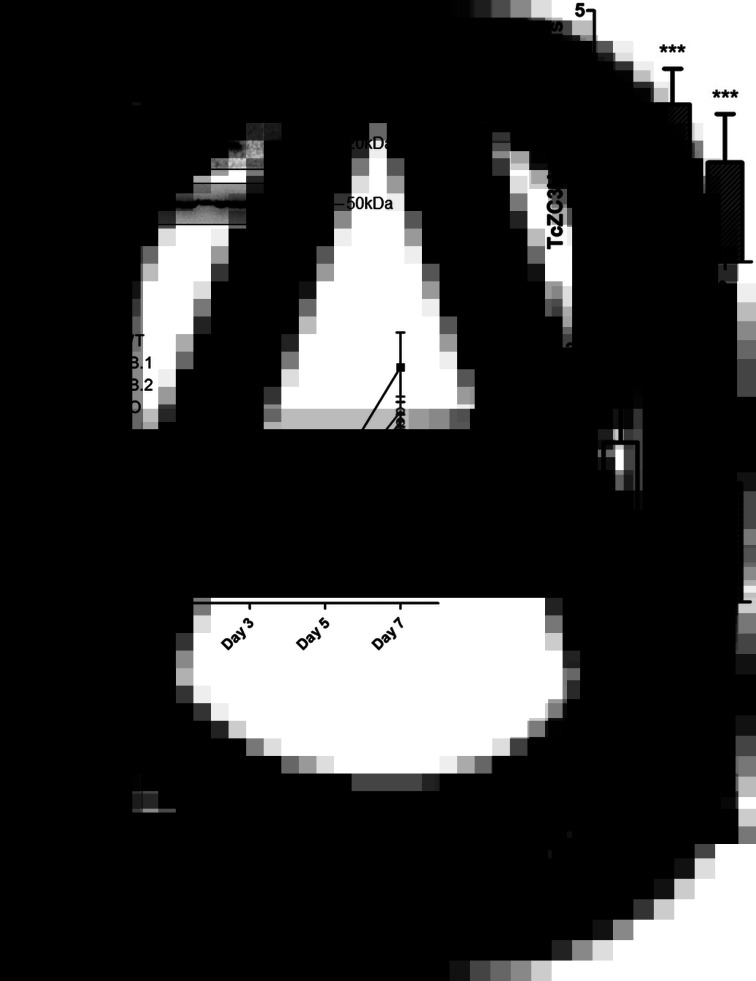
Re-expression of TcZC3H12 in KO mutants partially restores growth and differentiation phenotypes. (A) Western blot with total parasite protein extract using anti-HA primary antibody. *β*-Tubulin was used as loading control. (B) RT-qPCR using specific primers for TcZC3H12 of WT and two addback clones TcZC3H12-HA (AB.1 and AB.2). (C) Growth curve and (D) percent of metacyclic trypomastigotes were evaluated *in vitro* assay as previously described (***P < 0.001).

To further test the role of this RBP, we transfected one of the TcZC3H12 KO cell lines with the pROCK expression vector (DaRocha *et al*., 2004) containing the complete TcZC3H12 coding region fused to an HA-tag and the puromycin-resistance gene. After selection in LIT medium containing puromycin, we generated two addback cloned cell lines re-expressing TcZC3H12, as shown by western blots with an anti-HA antibody and qPCR using primers designed for TcZC3H12 mRNA amplification (Fig. 6A–C). As shown in Fig. 6B, qPCR assays demonstrated that the two TcZC3H12 addback cell lines have about 10-fold higher levels of TcZC3H12 transcripts compared to WT epimastigotes. It is thus expected that these addback cell lines may behave more similarly to a TcZC3H12 over expressor parasite than with WT epimastigotes. Because we used the pROCK expression vector, which contains the TcZC3H12 coding sequence driven by the ribosomal RNA promoter to transfect the KO cell line, it is not surprising that we generated an addback that over expresses TcZC3H12. In addition of being over expressed, the TcZC3H12 mRNA is constitutively expressed in the addback cell lines due to the 5′ and 3′ processing signals from housekeeping genes flanking the TcZC3H12 gene inserted in the pROCK vector. When growth curves and metacyclogenesis rates of two TcZC3H12 addback cell lines were compared to WT parasites, a partial reversion of the growth defect phenotype was observed since only a small difference in cell density was observed on day 7 (Fig. 6C). Although there is a tendency towards a reduction in the percentage of metacyclic trypomastigotes, no statistically significant differences in metacyclogenesis rates between WT and addback clones were detected (Fig. 6D). These results are matched by the results observed when we analysed cell lines over expressing the TcZC3H12 gene generated after transfecting WT epimastigotes with a pROCK-TcZC3H12-HA construct (Fig. S2A). Similar to the qPCR data comparing WT and addback clones, transfection of pROCK-TcZC3H12-HA results in parasites expressing about 15-fold more TcZC3H12 transcripts than WT cells (Fig. S2B). Also similar to the results obtained with the addback clones, a tendency to a reduced percentage of metacyclic trypomastigotes was observed with TcZC3H12 overexpressors compared to WT parasites and no differences in the growth curve were observed between WT parasites and over expressing parasites as well as with parasites transfected with the empty pROCK vector (Fig. S2C and D). Taken together, these data indicate that, in contrast to TcZC3H12 KO parasites, which have increased metacyclogenesis rates, over expression of this RBP has little impact on growth or metacyclogenesis rates.

### Epimastigote-specific transcripts are targets of TcZC3H12

**Fig. 7.**
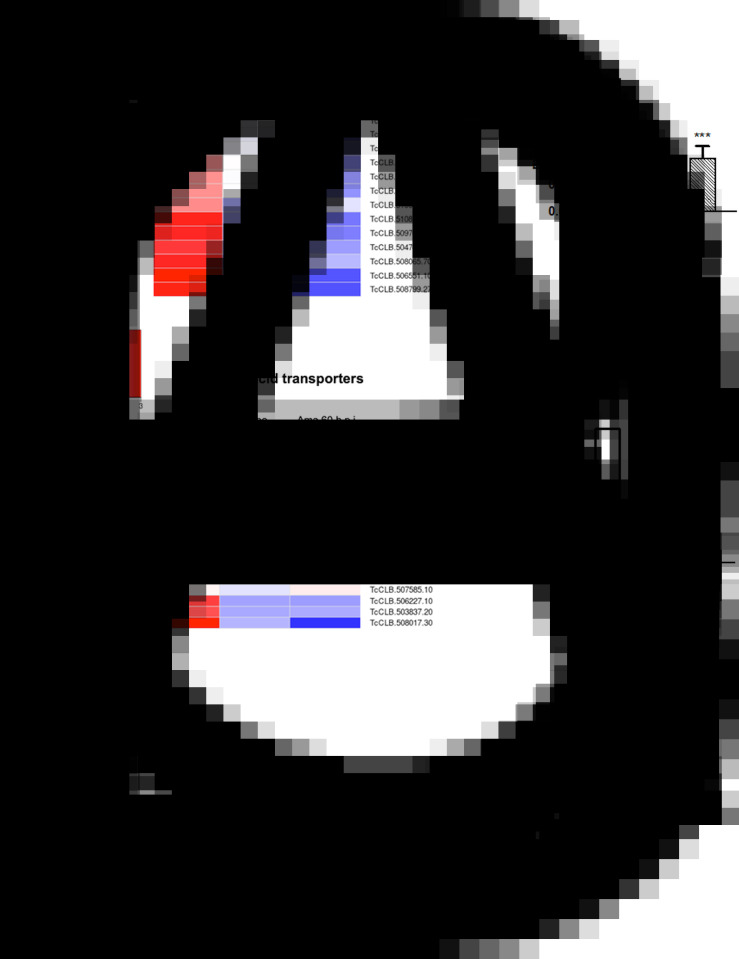
Potential targets of the TcZC3H12 among transcripts that are down-regulated in KO parasites. Comparative RNA-Seq analyses from epimastigotes, tissue culture-derived trypomastigotes, and intracellular amastigotes 60 h post-infection (hpi) were used to determine the global expression of *T. cruzi* CL Brener genes. Heat maps representing DE of mRNAs encoding the families: (A) PADs and (C) amino acid transporters in WT parasites. (B, D) RT-qPCRs to quantify PAD and amino acid transporters mRNA levels in WT epimastigotes (epi), WT trypomastigotes (tryp) and KO TcZC3H12 (KO.1) cell line using specific primers for one gene of the PAD family (TcCLB.506551.10) and one gene of the amino acid transporters (TcCLB.506153.10) family (***P < 0.001).

Based on the TcZC3H12 KO phenotype, observed both *in vitro* and *in vivo* we hypothesized that TcZC3H12 has a role in controlling parasite adaptation and differentiation in the insect vector. Previous studies based on proteomic and transcriptome analyses have shown that the parasite faces changes in gene expression when entering the stationary growth phase, which corresponds to the initial steps of metacyclogenesis (de Godoy *et al*., 2012; Berná *et al*., 2017; Avila *et al*., 2018; Santos *et al*., 2018). To investigate whether the absence of TcZC3H12 has an impact on global epimastigote gene expression, mRNA populations derived from biological triplicates of WT epimastigote cultures and duplicates of each TcZC3H12 KO cell line were sequenced using the MiSeq Illumina platform. After mapping the total number of reads to the *T. cruzi* CL Brener reference genome, mapped-sequencing data derived from all seven libraries were analysed using the PCA. As expected, the global transcriptome profiles of WT epimastigotes significantly differ from the profiles of both KO mutants (Fig. S3), i.e. disruption of the TcZC3H12 gene significantly affects gene expression in epimastigotes. To identify the genes that have their expression affected, we performed differential gene expression analyses using the DESeq2 package. Because the *T. cruzi* genome contains large gene families, various of them with more than 1000 copies (El-Sayed *et al*., 2005), for this DE analyses, all DE genes that belong to any of the six largest multigenic families (MASP, mucin, DGF-1, Trans-sialidase, RHS and GP63) were removed to avoid giving undue weight to those very repetitive transcripts. DE analyses identified 20 genes that are down-regulated in TcZC3H12 KO mutants compared to WT and 54 genes that are up-regulated in KO (top 12 DE genes are shown in Table 1; scores for all DE genes are shown in Table S7). Among the down-regulated genes, several members of the gene family encoding amino acid transporters were identified. Another gene family, encoding PADs, also has more than one member down-regulated in KO parasites compared to WT. Although their role in *T. cruzi* has not yet been investigated, PAD genes have been characterized in *T. brucei* as a gene family encoding carboxylate-transporters that convey differentiation signals in this parasite (Dean *et al*., 2009). As shown in the heat-maps obtained from RNA-Seq data comparing epimastigotes, trypomastigotes and amastigotes, PAD genes are up-regulated in epimastigote (Fig. 7A). Amino acid transporters, on the contrary, are highly expressed in both replicative forms, epimastigotes and amastigotes (Fig. 7C) (for more details on DE data, see Table S4). Similar results showing higher expression of PAD and amino acid transporter genes in epimastigotes were obtained from the RNA-Seq data previously described for the Dm28c (Smircich *et al*., 2015) and Y strains (Li *et al*., 2016).

**Table 1.** Differentially expressed genes in ZC3H12 KO epimastigotes compared to WT epimastigotes

Gene ID	Gene annotation	log 2FC	*P*_adj_ value
Up-regulated			
TcCLB.510131.40	Haloacid dehalogenase-like hydrolase, putative	1.446	9.35 × 10^−13^
TcCLB.507851.40	Hypothetical protein, conserved	1.319	2.2 × 10^−08^
TcCLB.511037.20	L-2-hydroxyglutarate dehydrogenase, mitochondrial, putative	1.189	6.72 × 10^−20^
TcCLB.506247.300	Hypothetical protein, conserved	1.058	1.8 × 10^−11^
TcCLB.504171.40	Mechanosensitive ion channel MscS, putative	1.048	6.95 × 10^−09^
Down-regulated			
TcCLB.506053.10	Amino acid transporter, putative	−1.882	2.12 × 10^−10^
TcCLB.503733.80	Hypothetical protein, conserved	−1.863	5.9 × 10^−20^
TcCLB.504173.40	Hypothetical protein	−1.848	2.14 × 10^−06^
TcCLB.506153.10	Amino acid transporter, putative	−1.785	3.6 × 10^−08^
TcCLB.504173.60	RING-variant domain containing protein, putative	−1.508	1.89 × 10^−08^
TcCLB.506551.10	Protein associated with differentiation 8, putative	−1.141	3.71 × 10^−12^
TcCLB.508799.270	Protein associated with differentiation 8, putative	−1.048	4.87 × 10^−08^

Only the top 12 DE genes are shown, a complete list is shown in Table S7.

We validated our transcriptome analysis by qPCR using primers designed to amplify one member of the amino acid transporter gene family (TcCLB.506153.10) and one member of the PAD (TcCLB.506551.10) family. qPCR amplification of RNA extracted from WT epimastigotes and tissue culture trypomastigotes as well as from TcZC3H12 KO epimastigotes confirmed that transcript levels for PAD and amino acid transporter genes are up-regulated in epimastigotes compared to trypomastigotes. Importantly, expression levels of both genes are significantly lower in TcZC3H12 KO epimastigotes than in WT epimastigotes (Fig. 7B and D).

**Fig. 8.**
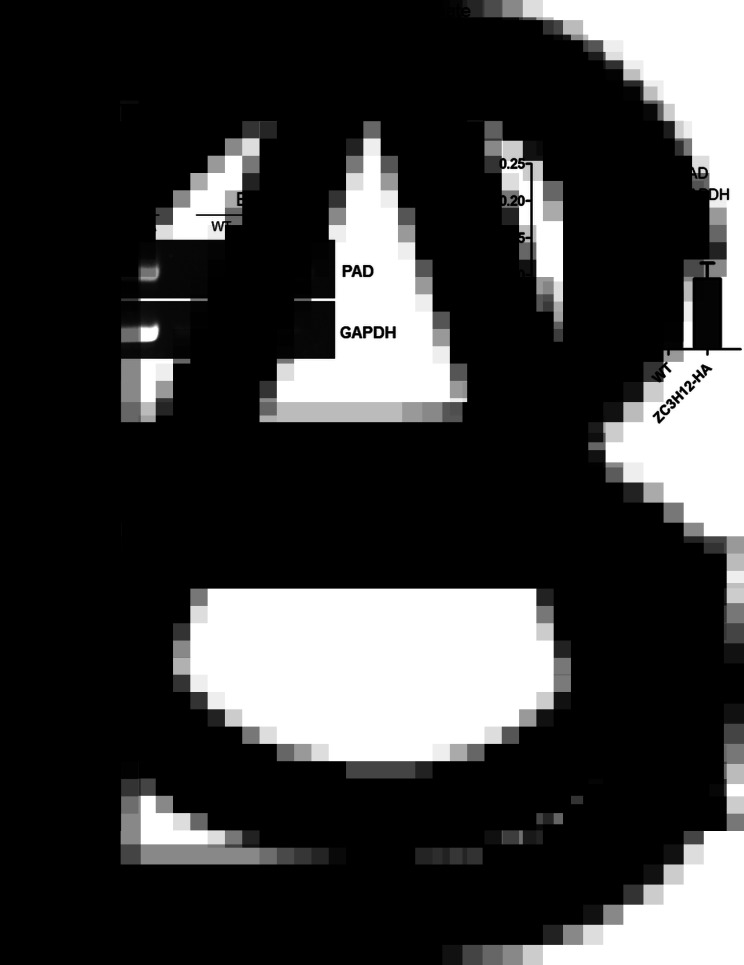
TcZC3H12 binds to PAD transcripts. Parasites expressing endogenous TcZC3H12 with HA tag were used in immunoprecipitation assay with beads conjugated to anti-HA antibodies. (A) Western blot with total protein extract of input, unbound and eluate fractions incubated with anti-HA antibodies. Total RNA was extracted from input and immunoprecipitated fractions, and one-step RT-PCR was performed using specific primers to the coding region of one member of the PAD family (TcCLB.506551.10) and GAPDH (B) Samples were run in an agarose gel and (C) intensity of the bands was quantified. Fold change of the intensity of immunoprecipitated *vs* input samples was calculate and plotted (results representative of two independent runs; *P < 0.05).

Due to the presence of the conserved HNPY sequence in the TcZC3H12 zinc finger protein, it can be predicted that this RBP interacts with the MKT1-PBP1 complex, which is involved with increasing mRNA abundance and translation rates in *T. brucei* (Singh *et al*., 2014). It is therefore less likely that genes that were found to be up-regulated in TcZC3H12 KO mutants may constitute direct targets of this RBP. Based on this assumption, we hypothesized that the mRNAs that are down-regulated in TcZC3H12 KO cell lines constitute targets of TcZC3H12 and that this RBP directly interacts with these mRNAs in epimastigotes. To test this hypothesis, WT epimastigotes and epimastigotes expressing an HA-tagged TcZC3H12 (inserted in the endogenous TcZC3H12 locus) were used in RNA immunoprecipitation assays. After UV crosslinking and cell lysis, sepharose beads conjugated to anti-HA antibody were used to immunoprecipitate the RNA–protein complexes. Total RNA purified from the immunoprecipitated beads as well as from input cell extracts prepared from both WT and TcZC3H12-HA transfected epimastigotes were used in RT-PCR assays. Figure 8A shows the results of western blot analysis performed with total protein extracts (input) as well as with immunoprecipitated fractions (eluate) and non-immunoprecipitated protein (unbound) prepared from WT and TcZC3H12-HA epimastigotes. A 20 kDa band corresponding to the tagged protein was observed only in the total cell extract and in the eluate fraction of transfected parasites. Using specific primers for the TcCLB.506551.10 gene, which encodes one member of the PAD family and for the GAPDH gene (TcCLB.506943.50), used as an endogenous control, we performed RT-PCR with the RNA extracted from total cell extracts and from immunoprecipitate (eluate) fractions. Gel images of the PCR products shown Fig. 8B confirmed that PAD transcripts are present only in the immunoprecipitate fractions of TcZC3H12-HA transfected epimastigotes, demonstrating that TcZC3H12 binds to at least one member of the PAD family. Densitometry analysis shown in Fig. 8C revealed a 20% enrichment of PAD transcripts in the eluate fraction derived from TcZC3H12-HA transfected epimastigotes compared to WT parasites, whereas GAPDH transcripts were found in the eluate fractions derived from both parasite cultures with roughly the same abundance. Taken together, the data presented here indicated that TcZC3H12 is an RBP that is abundantly expressed in epimastigotes and, through interactions with mRNAs that are up-regulated in this stage of the *T. cruzi* life cycle, affects the expression of parasite transcripts involved with epimastigote proliferation and differentiation.

## Discussion

*Trypanosoma cruzi* is a member of an early divergent group of eukaryotic organisms that is characterized by unique features with respect to genome organization and gene expression. Unlike most eukaryotes, transcription in Trypanosomatids is polycistronic and the processing of all mRNAs is dependent on coupled trans-splicing and polyadenylation reactions. Because of polycistronic transcription and the lack of individual promoters for every gene, regulation of gene expression must rely on RBPs, which are key elements involved in posttranscriptional control mechanisms. Previous works studying mechanisms controlling gene expression, mainly using *T. brucei* as a model organism, revealed the importance of stage-specific RBPs as trans-acting factors that modulate mRNA levels throughout the cell cycle, during different growth conditions and differentiation (Clayton, 2019; de Pablos *et al*., 2019). Our transcriptome analysis based on RNA-Seq data obtained from the three stages of the *T. cruzi* life cycle revealed that among 175 genes encoding *T. cruzi* RBPs, transcript levels of 29 RBP genes are up-regulated in the epimastigote insect stage compared to the mammalian stages (amastigotes and trypomastigotes). Among those, the gene encoding the zinc finger TcZC3H12 presented a 10-fold increase in transcript levels in epimastigotes compared to the other forms of the parasite. The same gene was also found to be highly up-regulated in epimastigotes of the Dm28c strain and, as shown here for the CL Brener strain, has decreased levels in metacyclic trypomastigotes (Smircich *et al*., 2015). Similarly, RNA-Seq data obtained from the Y strain of *T. cruzi* showed that TcZC3H12 has increased levels in epimastigotes compared to the other forms (Li *et al*., 2016). Altogether, RNA-Seq data and the results of immunofluorescence assays with HA-tagged parasites showed that TcZC3H12 is up-regulated in epimastigotes but has decreased expression in metacyclic trypomastigotes. The *T. brucei* orthologue, TbZC3H12, also encodes a CCCH-type zinc finger whose mRNA levels are increased in the late logarithmic growth phase of procyclic parasites (Fernández-Moya *et al*., 2014). Interestingly, both *T. cruzi* and *T. brucei* genes are syntenic but the *T. brucei* genome possess a second gene encoding a zinc finger protein, named TbZC3H13 that is absent in the *T. cruzi* genome (Ouna *et al*., 2012). The fact that this RBP is exclusive of members of the Trypanosomatid family, and its expression is associated with differentiation between life cycle stages present in the mammalian and insect hosts points towards a role as regulator of cell division and differentiation that is specific for these organisms. Studies on this type of RBP are highly relevant since Trypanosomatids belong to a group of early diverging eukaryotes, for which little is known about the mechanism involved in the regulation of gene expression during cell differentiation and development.

The two *T. brucei* orthologues, TbZC3H12 and TbZC3H13 encode cytosolic proteins that have similar expression levels in bloodstream and procyclic forms and are phosphorylated (Ouna *et al*., 2012). TbZC3H12 KO mutants were generated in bloodstream parasites and this gene deletion had no effects in parasite growth. RNA interference targeting TbZC3H12 and TbZC3H13 separately or in double knockdown also did not result in any significant phenotypical changes either in bloodstream or in procyclic forms. The authors speculated that, most likely, these proteins have a role that can only be observed in the life cycle stages found in natural hosts (Ouna *et al*., 2012). In contrast, our findings showed that TcZC3H12 has a regulatory role involved with epimastigote growth and differentiation in *T. cruzi*, since KO parasites have decreased growth rate and increased differentiation capacity, observed both *in vitro* and *in vivo*. We hypothesized that TcZC3H12 KO epimastigotes undergo earlier growth arrest and enter the stationary phase prematurely because TcZC3H12 positively regulates transcripts that are required for epimastigote proliferation. At the same time, if TcZC3H12 negatively regulates transcripts that are required for differentiation into metacyclic trypomastigotes, its absence may trigger metacyclogenesis during an early stage of the parasite development in the insect vector. The decreased levels in metacyclic trypomastigotes observed in parasites with the tagged protein corroborate this hypothesis.

The cytoplasmic localization of TcZC3H12 is also in accordance with its role as a protein that binds mRNAs and controls their steady state levels. The accumulation of this protein in granular structures, as shown by confocal microscopy, suggests that it might be part of ribonucleoprotein (RNPs) involved with mRNA storage. Experiments comparing the dynamics of association of TcZC3H12 with its target mRNAs and RNP granules during epimastigote growth and differentiation would provide further evidence for the role of this RBP. Although the mechanisms behind the formation of these cytoplasmic RNP granules as well as their function are not yet fully understood, several studies have showed that, both in *T. brucei* and *T. cruzi*, these granules are formed in response to nutritional stress and contain proteins homologous to those present in P bodies and stress granules from metazoan organisms (Cassola *et al*., 2007; Holetz *et al*., 2007, 2010; Romagnoli *et al*., 2020).

The presence of the conserved HNPY motif suggesting an interaction between TcZC3H12 and the MKT1-PBP1 complex also points towards a role of this zinc finger RBP as a factor involved with mRNA stabilization in *T. cruzi* epimastigotes. MKT1 has been shown to interact with multiple RBPs and other proteins involved in RNA regulation, acting as a master regulator of mRNA expression in *T. brucei* (Singh *et al*., 2014; Melo do Nascimento *et al*., 2020). A putative interaction between MKT1 and TbZC3H12 in *T. brucei* was described by Lueong *et al*. (2016), who also showed that the TcZC3H12 orthologue acts as a positive regulator of gene expression in procyclic parasites.

Aiming at identifying targets of the TcZC3H12 RBP, we analysed changes that occurred in the transcriptome of TcZC3H12 KO mutants. We identified 74 genes with altered expression, 20 of them showing down-regulation in the KO mutants compared to WT epimastigotes. For one member of the PAD gene family and one member of the amino acid transporter, the decreased levels of mRNA in KO epimastigotes were confirmed through RT-qPCR. When comparing epimastigotes in different growth phases, changes in gene expression were described as clusters of up- and down-regulated genes (Santos *et al*., 2018), as well as in differences in the proteome (de Godoy *et al*., 2012; Avila *et al*., 2018) and metabolome (Barisón *et al*., 2017). During the initial metacyclogenesis stages, significant morphological changes of epimastigotes, which include the position of the flagellar basal body (Avila *et al*., 2018; Gonçalves *et al*., 2018) are easily observed under the microscope. Also, major changes in the metabolism of epimastigotes are triggered by culture conditions that mimics the conditions found in the insect hindgut (Shaw *et al*., 2016), which include changes in glucose levels and amino acid composition (De Lima *et al*., 2008). The fact that we have identified amino acid transporters as part of the group of epimastigote-specific genes that were down-regulated in TcZC3H12 KO parasites is in agreement with data from the literature that showed that amino acids are the main source of energy for epimastigotes and, as a consequence, expression of these transporters are essential for epimastigote proliferation (Silber *et al*., 2005). Genes that are up-regulated in the KO epimastigotes may not be direct targets of TcZC3H12 and their increased expression may result from other changes in metabolic pathways that have occurred in the absence of this RBP. High-throughput sequencing of immunoprecipitated mRNAs using RIP-Seq protocols will provide essential information regarding not only the identification of all mRNA targets of TcZC3H12, but also the regulatory networks involving this RBP.

For at least one member of the PAD gene family, RNA co-immunoprecipitation assay allowed us to show that TcZC3H12 interacts with transcripts that are up-regulated in epimastigotes. These interactions may not be direct but may occur within a complex formed between TcZC3H12 and other proteins. Orthologues of PAD genes have been characterized in *T. brucei* as transmembrane proteins encoding carboxylate-transporters that are required for the perception of the differentiation signal mediated by citrate or *cis*-aconitate. TbPAD1 is expressed only by stumpy trypomastigotes, which can survive and differentiate inside the tsetse fly after a blood meal. TbPAD2, on the contrary, is highly expressed in procyclics and both proteins are not expressed by slender trypomastigotes that replicate in the mammalian bloodstream (Dean *et al*., 2009). Members of this gene family are thermoregulated in *T. brucei* (Dean *et al*., 2009) and in *Leishmania major* (Rastrojo *et al*., 2019). In *Trypanosoma congolense*, PAD expression was shown to be up-regulated in parasites located in the cardia (procyclics) of infected *Glossina morsitans* and down-regulated in to parasites located in the proboscis (metacyclic trypomastigotes) (Awuoche *et al*., 2018). These data corroborate the role of PAD proteins required for a successful transition between insect and mammalian hosts. Although two recently described zinc finger proteins (TbZC3H20 and TbZC3H21) were identified as regulatory factors that bind hundreds of mRNAs in *T. brucei* procyclic forms and depletion of TbZC3H20 causes a decrease in PAD1 expression, in contrast to the results shown here, none of these *T. brucei* zinc finger proteins were shown to bind PAD transcripts (Liu *et al*., 2020). Considering the importance of this gene family to convey differentiation signal in trypanosomes, it is highly relevant to further investigate the role TcZC3H12 as an RBP that controls PAD expression in *T. cruzi*.

Further studies are necessary to validate a model that proposes a regulatory role of TcZC3H12 during *T. cruzi* epimastigote cell division and differentiation. Sequencing the RNA population that is present in immunoprecipitation complexes containing this RBP under different growth conditions would provide a more detailed picture of its target transcripts whereas mass spectrometry analyses will allow identifying other parasite proteins that interact with TcZC3H12. Half-life measurements of its target transcripts may also shed some light on the regulatory mechanisms involving this RBP. These studies are highly needed for a deeper understanding of basic aspects of the biology of a protozoan parasite that still poses a significant threat to public health worldwide.

## Supplementary material

For supplementary material accompanying this paper visit https://doi.org/10.1017/S0031182020002176. click here to view supplementary material
