# Supplementary material for: A Trypanosoma cruzi zinc finger protein that is implicated in the control of epimastigote-specific gene expression and metacyclogenesis
Source: Parasitology. 2020 Nov 16;148(10):1171–85. doi: 10.1017/S0031182020002176 (PMC8312218; doi:10.1017/S0031182020002176)
Supplement: Supplementary file 1 [file S0031182020002176sup001.zip › S0031182020002176sup005.docx]

**Table S2. Annotated genes corresponding to RNA Binding Proteins identified in the CL Brener genome.**

| Gene ID | TriTrypDB anotation | Domain |
| --- | --- | --- |
| TcCLB.507711.40 | RNA-binding protein | RRM |
| TcCLB.424123.40 |  |  |
| TcCLB.469785.40 | RNA-binding protein | RRM |
| TcCLB.507025.50 |  |  |
| TcCLB.507025.60 | RNA-binding protein | RRM |
| TcCLB.503419.50 | MRB1-associated protein | RRM |
| TcCLB.509105.90 |  |  |
| TcCLB.503619.20 | Hypothetical protein | RRM |
| TcCLB.511647.40 |  |  |
| TcCLB.503683.11 | Double RNA binding domain protein 6A | RRM |
| TcCLB.509999.140 |  |  |
| TcCLB.503683.30 | RNA-binding protein | RRM |
| TcCLB.509999.120 |  |  |
| TcCLB.509581.50 | Hypothetical protein | RRM |
| TcCLB.503709.10 |  |  |
| TcCLB.508569.90 | Hypothetical protein | RRM |
| TcCLB.503733.50 |  |  |
| TcCLB.503897.90 | Hypothetical protein | RRM |
| TcCLB.509561.110 |  |  |
| TcCLB.507093.63 | RNA-binding protein | RRM |
| TcCLB.503917.7 |  |  |
| TcCLB.507037.20 | RNA-binding protein | RRM |
| TcCLB.508707.80 |  |  |
| TcCLB.508209.39 | Hypothetical protein | RRM |
| TcCLB.503919.30 |  |  |
| TcCLB.509591.50 | RNA-binding protein 34 | RRM |
| TcCLB.503999.90 |  |  |
| TcCLB.504005.6 | RNA-binding protein | RRM |
| TcCLB.511481.55 |  |  |
| TcCLB.504071.80 | Nuclear cap binding protein | RRM |
| TcCLB.507089.70 | RNA-binding protein | RRM |
| TcCLB.504085.30 |  |  |
| TcCLB.511109.130 | Heterogeneous nuclear ribonucleoprotein H/F | RRM |
| TcCLB.504157.10 |  |  |
| TcCLB.504165.20 | Hypothetical protein | RRM |
| TcCLB.508981.20 |  |  |
| TcCLB.508145.30 | RNA-binding protein | RRM |
| TcCLB.504243.10 |  |  |
| TcCLB.504431.90 | U2 small nuclear ribonucleoprotein B | RRM |
| TcCLB.507951.140 |  |  |
| TcCLB.505007.10 | RNA-binding protein | RRM |
| TcCLB.510007.30 |  |  |
| TcCLB.505165.10 | RNA-binding protein | RRM |
| TcCLB.506399.40 | Hypothetical protein | RRM |
| TcCLB.509243.20 |  |  |
| TcCLB.506425.60 | Hypothetical protein | RRM |
| TcCLB.506565.12 | RNA-binding protein | RRM |
| TcCLB.511837.129 |  |  |
| TcCLB.506565.4 | RNA-binding protein | RRM |
| TcCLB.506565.8 | RNA-binding protein | RRM |
| TcCLB.506625.70 | RNA-binding protein | RRM |
| TcCLB.506649.80 | Double RNA binding domain protein 3 | RRM |
| TcCLB.508349.39 |  |  |
| TcCLB.506825.10 | Double RNA binding domain protein 12 | RRM |
| TcCLB.506681.10 |  |  |
| TcCLB.506693.30 | RNA-binding protein 6 | RRM |
| TcCLB.508153.680 |  |  |
| TcCLB.506795.10 | RNA-binding protein | RRM |
| TcCLB.509937.60 |  |  |
| TcCLB.506797.120 | Hypothetical protein | RRM |
| TcCLB.511907.100 |  |  |
| TcCLB.509167.30 | Hypothetical protein | RRM |
| TcCLB.506831.30 |  |  |
| TcCLB.506925.400 | Hypothetical protein | RRM |
| TcCLB.506989.100 | Triple RNA binding domain protein 3 | RRM |
| TcCLB.510149.140 |  |  |
| TcCLB.507093.220 | RNA-binding protein | RRM |
| TcCLB.507093.229 | U-rich RNA-binding protein UBP-2 | RRM |
| TcCLB.507093.250 | RNA-binding protein | RRM |
| TcCLB.508213.40 | Hypothetical protein | RRM |
| TcCLB.507515.40 |  |  |
| TcCLB.508213.20 | Hypothetical protein | RRM |
| TcCLB.507515.60 |  |  |
| TcCLB.507611.300 | U1A small nuclear ribonucleoprotein | RRM |
| TcCLB.507723.120 |  |  |
| TcCLB.507873.30 | Double RNA binding domain protein 7 | RRM |
| TcCLB.510689.60 |  |  |
| TcCLB.507885.10 | RNA-binding protein | RRM |
| TcCLB.507993.140 | RNA-binding protein 29 | RRM |
| TcCLB.511277.580 |  |  |
| TcCLB.508145.10 | RNA-binding protein | RRM |
| TcCLB.508145.20 | RNA-binding protein | RRM |
| TcCLB.510143.80 | RNA-binding protein | RRM |
| TcCLB.508409.270 |  |  |
| TcCLB.510755.120 | RNA-binding protein | RRM |
| TcCLB.508413.50 |  |  |
| TcCLB.508461.320 | U5 snRNA-associated splicing factor | RRM |
| TcCLB.510877.150 | Eukaryotic translation initiation factor 3 subunit g | RRM |
| TcCLB.508689.20 |  |  |
| TcCLB.508837.140 | u1 small nuclear ribonucleoprotein 70 kDa | RRM |
| TcCLB.511383.30 |  |  |
| TcCLB.511277.200 | RNA-binding protein | RRM |
| TcCLB.511367.60 | Lupus La protein homolog | RRM |
| TcCLB.511127.10 | RNA-binding protein 5 | RRM |
| TcCLB.511481.70 |  |  |
| TcCLB.508879.80 | Hypothetical protein | RRM |
| TcCLB.424195.9 | RNA-binding protein 4 | RRM |
| TcCLB.508901.20 |  |  |
| TcCLB.508989.30 | RNA-binding protein | RRM |
| TcCLB.509569.120 |  |  |
| TcCLB.509965.180 | RNA-binding protein | RRM |
| TcCLB.509053.179 |  |  |
| TcCLB.509055.10 | RNA-binding protein | RRM |
| TcCLB.511621.50 | RNA-binding protein | RRM |
| TcCLB.509317.60 |  |  |
| TcCLB.510507.50 | RNA-binding protein | RRM |
| TcCLB.509461.100 |  |  |
| TcCLB.510105.33 | RNA-binding protein | RRM |
| TcCLB.509715.23 |  |  |
| TcCLB.511741.40 | RNA-binding protein | RRM |
| TcCLB.511517.70 |  |  |
| TcCLB.511727.160 | Polypyrimidine tract-binding protein | RRM |
| TcCLB.511727.190 | Hypothetical protein | RRM |
| TcCLB.511727.270 | RNA-binding protein | RRM |
| TcCLB.511727.290 | RNA-binding protein | RRM |
| TcCLB.511871.110 | Hypothetical protein | RRM |
| TcCLB.511863.20 |  |  |
| TcCLB.511837.138 | RNA-binding protein | RRM |
| TcCLB.510311.50 | Hypothetical protein | RRM |
| TcCLB.510265.40 |  |  |
| TcCLB.510747.80 | Double RNA binding domain protein 9 | RRM |
| TcCLB.510657.160 |  |  |
| TcCLB.506435.120 | ATP-dependent RNA helicase * | RRM |
| TcCLB.506265.100 |  |  |
| TcCLB.511003.30 | Mitochondrial RNA binding complex 1 subunit * | RRM |
| TcCLB.503715.10 | Splicing factor TSR1, putative * | RRM |
| TcCLB.509607.30 |  |  |
| TcCLB.511181.70 | Hypothetical protein, conserved * | RRM |
| TcCLB.508299.89 |  |  |
| TcCLB.510741.40 | Hypothetical protein, conserved * | RRM |
| TcCLB.510661.230 |  |  |
| TcCLB.509167.140 | RNA-binding protein 42 * | RRM |
| TcCLB.485683.10 |  |  |
| TcCLB.506885.70 | Polyadenylate-binding protein 1 | RRM/PABP |
| TcCLB.508461.140 |  |  |
| TcCLB.503989.10 | Zinc finger protein 2 | Zinc Finger |
| TcCLB.401469.10 |  |  |
| TcCLB.411427.10 | Hypothetical protein | Zinc Finger |
| TcCLB.506859.204 | Hypothetical protein | Zinc Finger |
| TcCLB.503567.9 |  |  |
| TcCLB.503577.20 | U2 splicing auxiliary factor | Zinc Finger |
| TcCLB.510943.60 |  |  |
| TcCLB.503795.10 | Hypothetical protein | Zinc Finger |
| TcCLB.506679.10 |  |  |
| TcCLB.503897.150 | Hypothetical protein | Zinc Finger |
| TcCLB.509561.39 |  |  |
| TcCLB.507089.30 | Zinc finger protein | Zinc Finger |
| TcCLB.504085.70 |  |  |
| TcCLB.511577.30 | Hypothetical protein | Zinc Finger |
| TcCLB.504797.110 |  |  |
| TcCLB.504929.5 | Zinc finger CCCH domain containing protein 11 | Zinc Finger |
| TcCLB.507305.40 |  |  |
| TcCLB.510295.59 | Hypothetical protein | Zinc Finger |
| TcCLB.510297.10 | Hypothetical protein | Zinc Finger |
| TcCLB.506009.10 |  |  |
| TcCLB.506127.20 | mRNA export factor MEX67 | Zinc Finger |
| TcCLB.506211.60 | RNA-binding protein | Zinc Finger |
| TcCLB.508895.60 |  |  |
| TcCLB.506211.70 | RNA-binding protein | Zinc Finger |
| TcCLB.508895.50 |  |  |
| TcCLB.510101.200 | Zinc finger ccch and cchc domain-containing protein | Zinc Finger |
| TcCLB.506297.130 |  |  |
| TcCLB.506733.140 | Hypothetical protein | Zinc Finger |
| TcCLB.509229.90 |  |  |
| TcCLB.506739.99 | Zinc finger (CCCH type) protein | Zinc Finger |
| TcCLB.510819.119 |  |  |
| TcCLB.510759.100 | Hypothetical protein | Zinc Finger |
| TcCLB.506999.120 |  |  |
| TcCLB.509551.60 | Hypothetical protein | Zinc Finger |
| TcCLB.507007.77 |  |  |
| TcCLB.508271.4 | Hypothetical protein | Zinc Finger |
| TcCLB.506859.80 | Hypothetical protein | Zinc Finger |
| TcCLB.511815.50 |  |  |
| TcCLB.506859.230 | Hypothetical protein | Zinc Finger |
| TcCLB.511817.10 |  |  |
| TcCLB.506859.240 | Hypothetical protein | Zinc Finger |
| TcCLB.511817.20 |  |  |
| TcCLB.510427.10 | Hypothetical protein | Zinc Finger |
| TcCLB.506883.120 |  |  |
| TcCLB.506885.200 | Hypothetical protein | Zinc Finger |
| TcCLB.510729.220 |  |  |
| TcCLB.506885.204 | Hypothetical protein | Zinc Finger |
| TcCLB.510729.210 |  |  |
| TcCLB.506931.4 | Hypothetical protein | Zinc Finger |
| TcCLB.510131.44 |  |  |
| TcCLB.506933.50 | Hypothetical protein | Zinc Finger |
| TcCLB.506945.210 | Zinc finger CCCH domain-containing protein 47 | Zinc Finger |
| TcCLB.503453.20 | Hypothetical protein | Zinc Finger |
| TcCLB.506977.110 | Hypothetical protein | Zinc Finger |
| TcCLB.510351.80 | Hypothetical protein | Zinc Finger |
| TcCLB.507601.80 |  |  |
| TcCLB.507625.70 | Hypothetical protein | Zinc Finger |
| TcCLB.507787.140 |  |  |
| TcCLB.511867.10 | Hypothetical protein | Zinc Finger |
| TcCLB.507775.10 |  |  |
| TcCLB.507831.20 | Hypothetical protein | Zinc Finger |
| TcCLB.511263.50 |  |  |
| TcCLB.507831.30 | Hypothetical protein | Zinc Finger |
| TcCLB.511263.40 |  |  |
| TcCLB.507831.40 | Hypothetical protein | Zinc Finger |
| TcCLB.511263.30 |  |  |
| TcCLB.508215.10 | Hypothetical protein | Zinc Finger |
| TcCLB.508879.10 |  |  |
| TcCLB.508241.90 | Hypothetical protein | Zinc Finger |
| TcCLB.511151.20 |  |  |
| TcCLB.508357.9 | Hypothetical protein | Zinc Finger |
| TcCLB.508355.330 |  |  |
| TcCLB.510143.120 | Hypothetical protein | Zinc Finger |
| TcCLB.508409.310 |  |  |
| TcCLB.508693.40 | Hypothetical protein | Zinc Finger |
| TcCLB.508831.66 | Hypothetical protein | Zinc Finger |
| TcCLB.511671.130 |  |  |
| TcCLB.509805.240 | Hypothetical protein | Zinc Finger |
| TcCLB.511267.20 |  |  |
| TcCLB.509805.230 | Hypothetical protein | Zinc Finger |
| TcCLB.511267.24 |  |  |
| TcCLB.511467.4 | Hypothetical protein | Zinc Finger |
| TcCLB.509231.39 | Hypothetical protein | Zinc Finger |
| TcCLB.509719.69 |  |  |
| TcCLB.509233.210 | Hypothetical protein | Zinc Finger |
| TcCLB.463297.10 | Hypothetical protein | Zinc Finger |
| TcCLB.509395.40 |  |  |
| TcCLB.511039.39 | Hypothetical protein | Zinc Finger |
| TcCLB.509791.90 |  |  |
| TcCLB.511555.40 | cleavage and polyadenylation specificity factor 30 kDa subunit | Zinc Finger |
| TcCLB.510219.30 |  |  |
| TcCLB.511511.3 | Zinc finger protein ZFP1 | Zinc Finger |
| TcCLB.511511.6 | Zinc finger protein ZFP1 | Zinc Finger |
| TcCLB.511735.40 | Hypothetical protein | Zinc Finger |
| TcCLB.511521.20 |  |  |
| TcCLB.511735.88 | Hypothetical protein | Zinc Finger |
| TcCLB.511807.130 | Hypothetical protein | Zinc Finger |
| TcCLB.511807.160 | Hypothetical protein | Zinc Finger |
| TcCLB.511867.60 | Hypothetical protein | Zinc Finger |
| TcCLB.507611.398 | RNA editing complex protein MP63, putative * | Zinc Finger |
| TcCLB.506247.30 |  |  |
| TcCLB.506357.69 | Hypothetical protein, conserved * | Zinc Finger |
| TcCLB.511277.39 |  |  |
| TcCLB.506533.70 | hypothetical protein, conserved * | Zinc Finger |
| TcCLB.509395.10 |  |  |
| TcCLB.510149.70 | Zinc-finger double-stranded RNA-binding, putative * | Zinc Finger |
| TcCLB.506989.20 |  |  |
| TcCLB.509443.20 | Mitochondrial RNA binding protein 1, putative * | Zinc Finger |
| TcCLB.507017.140 |  |  |
| TcCLB.510857.40 | RNA-editing complex protein, putative * | Zinc Finger |
| TcCLB.509611.110 |  |  |
| TcCLB.510743.20 | Hypothetical protein, conserved * | Zinc Finger |
| TcCLB.510659.279 | Nucleic acid binding protein, putative * |  |
| TcCLB.510687.20 | Zinc knuckle, putative * | Zinc Finger |
| TcCLB.397937.10 | Pumilio/PUF RNA binding protein 1 | PUF |
| TcCLB.508625.160 |  |  |
| TcCLB.508787.30 | Pumilio/PUF RNA binding protein 3 | PUF |
| TcCLB.468005.9 |  |  |
| TcCLB.503757.30 | Hypothetical protein | PUF |
| TcCLB.503719.39 |  |  |
| TcCLB.506563.10 | Pumilio/PUF RNA binding protein 9 | PUF |
| TcCLB.503869.40 |  |  |
| TcCLB.506773.130 | Pumilio-repeat, RNA-binding protein | PUF |
| TcCLB.508799.70 |  |  |
| TcCLB.508479.120 | Pumilio/PUF RNA binding protein 8 | PUF |
| TcCLB.507049.199 | Pumilio protein | PUF |
| TcCLB.508577.100 | Pumilio/PUF RNA binding protein 5 | PUF |
| TcCLB.507521.110 |  |  |
| TcCLB.507831.110 | Pumilio/PUF RNA binding protein 2 | PUF |
| TcCLB.511261.120 |  |  |
| TcCLB.511715.100 | Pumilio/PUF RNA binding protein 7 | PUF |
| TcCLB.508445.99 |  |  |
| TcCLB.510073.30 | Pumilio/PUF RNA binding protein 4 | PUF |
| TcCLB.509399.190 |  |  |
| TcCLB.509759.19 | Pumilio/PUF RNA binding protein 1 | PUF |
| TcCLB.510125.10 | Pumilio/PUF RNA binding protein 6 | PUF |
| TcCLB.510121.110 | Hypothetical protein, conserved * | PUF |
| TcCLB.506605.100 | Hypothetical protein * | KH |
| TcCLB.511239.90 |  |  |
| TcCLB.506625.200 | Ribosomal RNA processing protein 40, putative * | KH |
| TcCLB.510595.20 | KH domain, putative * | KH |
| TcCLB.507895.150 |  |  |
| TcCLB.510167.29 | Ribosomal RNA processing protein 4, putative * | KH |
| TcCLB.508859.109 |  |  |
| TcCLB.508717.40 | Branch point binding protein, putative * | KH |
| TcCLB.504079.10 |  |  |
| TcCLB.506943.120 | Hypothetical protein, conserved * | KH |
| TcCLB.509065.130 |  |  |
| TcCLB.507011.50 | Pre-rRNA-processing protein PNO1, putative * | KH |
| TcCLB.507005.70 |  |  |
| TcCLB.504089.60 | Hypothetical protein | Alba |
| TcCLB.504001.10 |  |  |
| TcCLB.504089.70 | Hypothetical protein | Alba |
| TcCLB.504001.20 |  |  |
| TcCLB.510877.30 | Hypothetical protein | Alba |
| TcCLB.510877.40 | Hypothetical protein | Alba |
| TcCLB.508489.20 | SAM domain (Sterile alpha motif), putative * | SAM |
| TcCLB.511495.20 |  |  |
| TcCLB.511517.140 | Hypothetical protein * | SAM |
| TcCLB.506743.4 | Eukaryotic translation initiation factor 1A, putative * | S1 |
| TcCLB.503945.10 |  |  |
| TcCLB.511367.240 | PIWI-like protein * | PIWI |

*Genes listed after last search for RBPs.
