# Supplementary material for: A Trypanosoma cruzi zinc finger protein that is implicated in the control of epimastigote-specific gene expression and metacyclogenesis
Source: Parasitology. 2020 Nov 16;148(10):1171–85. doi: 10.1017/S0031182020002176 (PMC8312218; doi:10.1017/S0031182020002176)
Supplement: Supplementary file 1 [file S0031182020002176sup001.zip › S0031182020002176sup006.docx]

**Table S3. Primer and oligonucleotide sequences**

| Primer | Sequence |
| --- | --- |
| For_TcZC3H12_XbaI | 5’tctagaATGCCCAAGGCCAACAAG’3 |
| Rev_TcZC3H12::HA_XhoI | 5’ctcgagTTAAGCGTAATCTGGTACGTCGTATGGGTATTCGTAATATGTAGAGAGAG’3 |
| For_5’KO_TcZC3H12_HindIII | 5’aagcttGGAGAACTTAAGGCATTCAT3’ |
| Rev_5’KO_TcZC3H12_SacI | 5’gagctcCGTTCAGAGAACTCTCTG3’ |
| For_3’KO_TcZC3H12_XhoI | 5’ctcgagGAGTTGTTGGGTTTGAGC3’ |
| Rev_3’KO_TcZC3H12_XbaI | 5'tctagaCCTGTAGGAAAGCAAAGCCG3’ |
| sgTcZC3H12 | 5’GGAGGCCGGAGAATTGTAATACGACTCACTATAGGACTTCCGTGTTGGACTGAGTCGTTTTAGTACTCTGGAAACAGAATC3’ |
| sgSpCas9_REV | 5’AAAAAAGCACCGACTCGGGCCACTT3’ |
| For_TcZC3H12 | 5’AACTCGCGTTACAAGGAGGG3’ |
| Rev_TcZC3H12 | 5’TCTCCCCCGTAAACCGACCT3’ |
| For_RPL9_qPCR | 5’TGACAACTCGACCATCAACA3’ |
| Rev_RPL9_qPCR | 5’GGCGAAGCGAATCTTAAAAC3’ |
| For_TcZC3H12_qPCR | 5’GGCGAAGCGAATCTTAAAAC3’ |
| Rev_TcZC3H12_qPCR | 5’GGCCTCACGCTCATCTAGTC3’ |
| For_PAD_qPCR | 5’GGTGGAGACGGACGTCGA3’ |
| Rev_PAD_qPCR | 5’TAGACAAAGCTGGCGTTG3’ |
| For_transporter_qPCR | 5’CTGCTGGGCAGCTTCTGT3’ |
| Rev_transporter_qPCR | 5’GACAACCGCAACCACACCAG3’ |
| For_Neo | 5’CGACCCTGCAGCCAATATGGGATCG3’ |
| Rev_Neo | 5’TCAGAAGAACTCGTCAAGAAGGCG3’ |
| For_Higro | 5’ATGAAAAAGCCTGAACTCACCGC3’ |
| For_CDS_PAD | 5’ATGACCGGCGAACAATTTG3’ |
| Rev_CDS_PAD | 5’TTACGGACGTGCCACCTT3’ |
| For_CDS_GAPDH | 5’ATGCCCATCAAGGTCGGTATC3’ |
| Rev_CDS_GAPDH | 5’CTACAACCTTGCCGAACGATC3’ |

*Lowercase letters indicate enzymes restriction sites

*Underlined letters represent HA epitope
