# Supplementary figures and images for: A Trypanosoma cruzi zinc finger protein that is implicated in the control of epimastigote-specific gene expression and metacyclogenesis
Source: Parasitology. 2020 Nov 16;148(10):1171–85. doi: 10.1017/S0031182020002176 (PMC8312218; doi:10.1017/S0031182020002176)

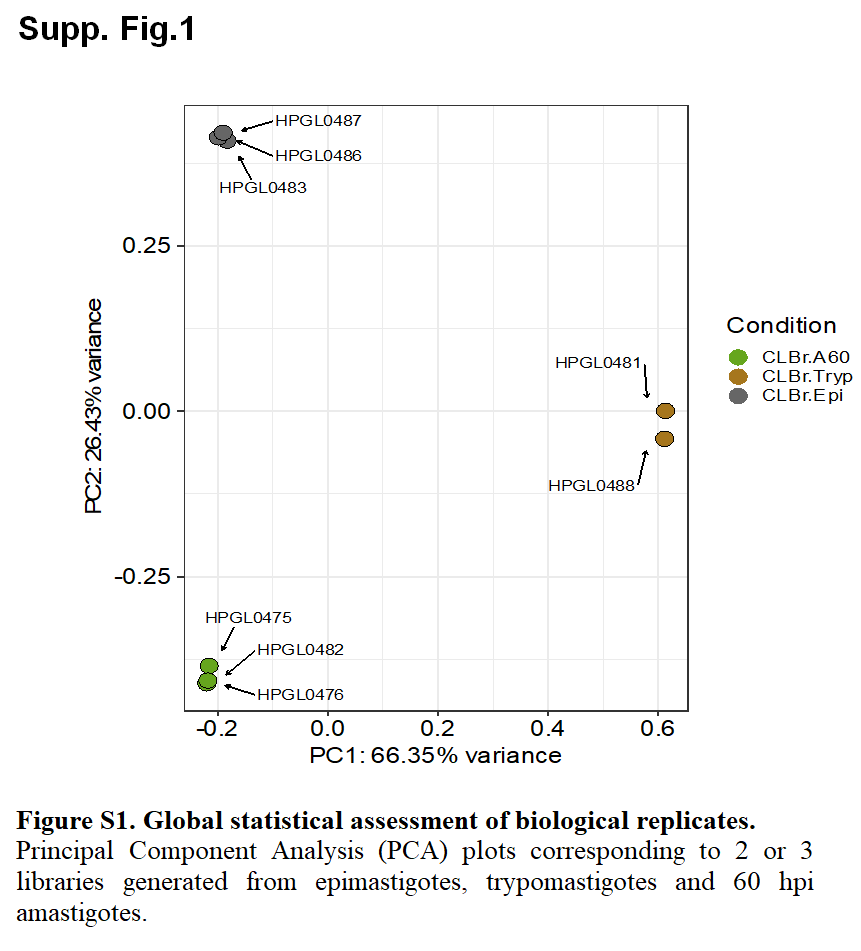

Supplement: Supplementary file 1 [file S0031182020002176sup001.zip › S0031182020002176sup001.tif]

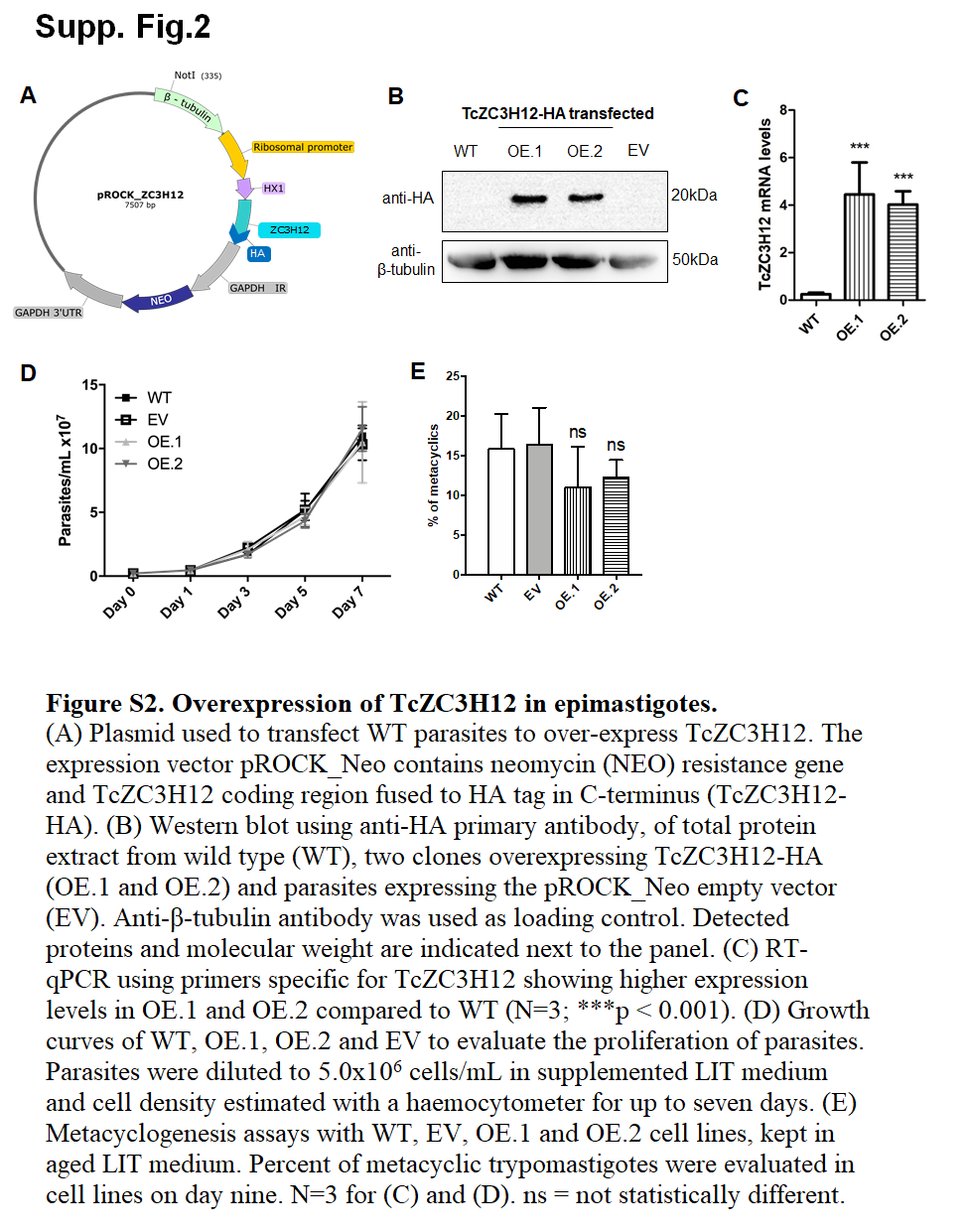

Supplement: Supplementary file 1 [file S0031182020002176sup001.zip › S0031182020002176sup002.tif]

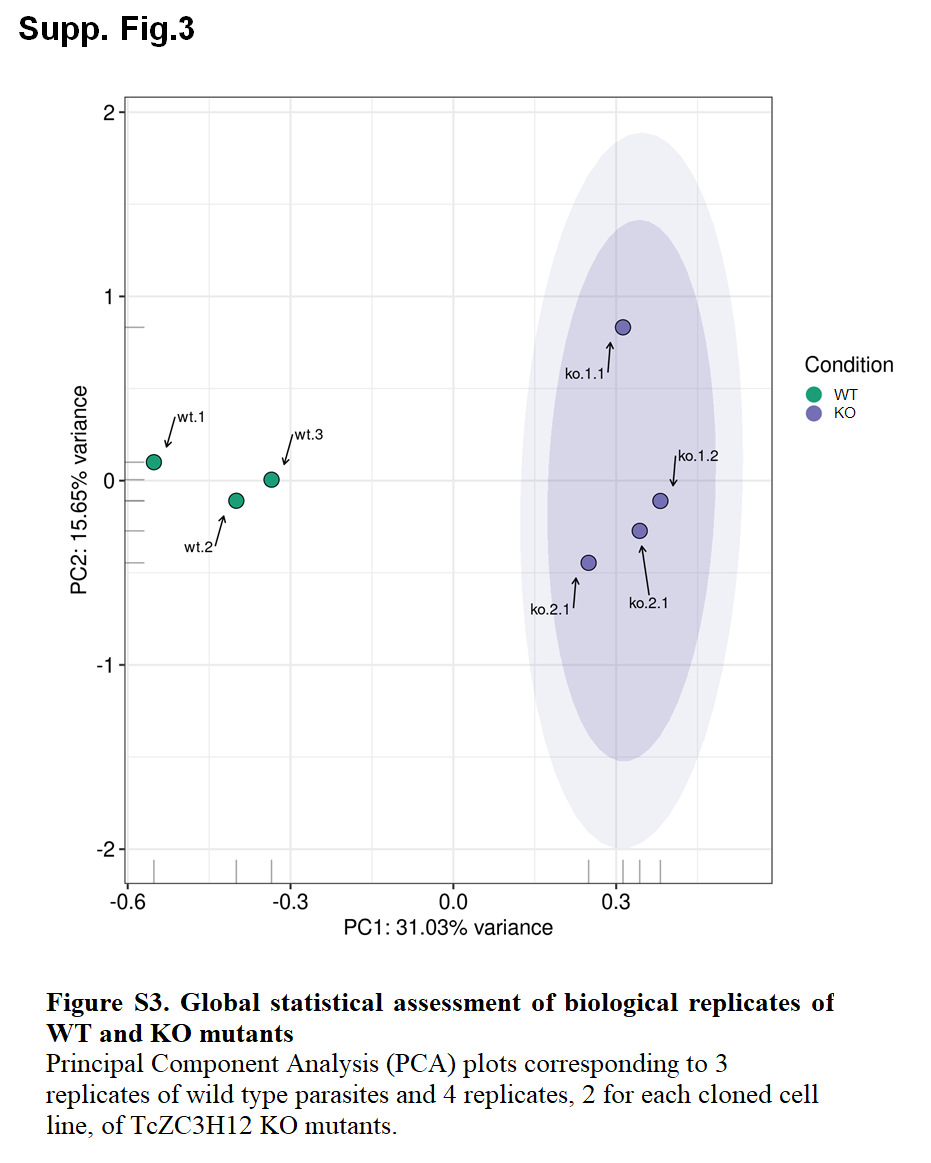

Supplement: Supplementary file 1 [file S0031182020002176sup001.zip › S0031182020002176sup003.tif]
